# The “minute diving beetles” of southern Australia – taxonomic revision of *Gibbidessus* Watts, 1978, with description of six new species (Coleoptera, Dytiscidae, Bidessini)

**DOI:** 10.3897/zookeys.975.55456

**Published:** 2020-10-12

**Authors:** Lars Hendrich, Chris H.S. Watts, Michael Balke

**Affiliations:** 1 SNSB – Zoologische Staatssammlung München, Münchhausenstraße 21, D – 81247 München, Germany; 2 South Australian Museum, North Terrace, Adelaide, South Australia 5000, Australia; 3 SNSB – Zoologische Staatssammlung München, Münchhausenstraße 21, D – 81247 München, Germany

**Keywords:** Key, mitochondrial DNA, new species, smallest epigean Australian Dytiscidae species, southern Australia, temporary wetlands, peatlands

## Abstract

Morphology and mitochondrial DNA sequence data are used to reassess the taxonomy of Australian diving beetles previously assigned to the genera *Uvarus* Guignot, 1939 and *Gibbidessus* Watts, 1978. *Gibbidessus* was described as a monotypic genus for *Gibbidessus chipi* Watts, 1978. The genus is significantly extended here. Based on molecular systematic evidence, *Uvarus pictipes* (Lea, 1899) is transferred to *Gibbidessus*. *Gibbidessus chipi* and *Gibbidessus pictipes***comb. nov.** are redescribed, and six new species are described: *Gibbiddessus atomus***sp. nov.** (SW Australia, Northcliffe area) [the smallest epigean diving beetle in Australia], *G. davidi***sp. nov.** (SW Australia), *G. drikdrikensis***sp. nov.** (Victoria), *G. kangarooensis***sp. nov.** (SA Kangaroo Island), *G. pederzanii***sp. nov.** (SW Australia, Nannup area), and G. *rottnestensis***sp. nov.** (SW Australia). Species are delineated using characters such as male genital structure and beetle size, shape and colour pattern. Mitochondrial Cox1 data for 27 individuals, representing five species, were generated, and revealed clusters congruent with the morphological evidence. *Gibbidessus* occur in southern Australia, with the centre of diversification in the isolated peat- and wetlands of SW Australia. All species occur in very shallow water of seasonal, exposed or half-shaded wetlands and flooded meadows.

## Introduction

With 726 described species the Bidessini belong to the most diverse tribes of the Dytiscidae (Nilsson and Hájek 2020). Bidessini genera have to date been justified mainly based on a diagnostic combination of structural features (Biström 1988; Miller and Short 2015), rather than apomorphies. This had to lead to the recognition of genera that render others paraphyletic (Balke and Ribera 2004). Some of these features, such as presence / absence of an elytral plica or occipital line, have been shown to vary within clades of closely related species (Balke et al. 2015). In this context, the use of phylogenetic reconstructions based on DNA sequence data offers a source of information that helps to delineate monophyletic entities (Hendrich and Balke 2009; Balke et al. 2013). In Australia, the situation is currently rather stable. Most Australian genera have been revised or will be revised in the near future (Balke and Ribera 2004; Watts 1978; Watts and Humphreys 2001, 2003, 2004, 2006, 2009; Watts and Leys 2005; Hendrich and Wang 2006; Hendrich and Balke 2009).

In this work we focus on the genus *Gibbidessus* Watts, 1978. These are widespread diving beetles of south-western and south-eastern Australia, but rarely collected, supposedly due to their small size. In fact, some of the species belong to the smallest epigean Australian Dytiscidae. We use molecular systematic evidence to redefine the genus and taxonomically treat all species now assigned to *Gibbidessus*, two known ones and six new species. We provide mitochondrial 3’ cox1 sequence data for five species.

## Materials and methods

**Material:** This study is based on the examination of 767 specimens. Types of the two previously known species were examined. Most of the specimens were collected in the past 25 years by LH and CHS Watts. Additional material was collected by Melita Pennifold of the Department of Parks and Wildlife in many parts of south-western Australia, and by Australian Water Quality in South Australia. Furthermore, the authors have studied all available specimens stored in relevant Australian museums.

**Descriptions:** Beetles were studied with a Leica M205C dissecting microscope at 10–100×. Male genitalia were studied and figured in dry condition. The terminology to denote the orientation of the genitalia follows Miller & Nilsson (2003). Abbreviations used in the text are: **TL** (total length), **TL-H** (total length without head), and **MW** (maximum width). Label data of type material are cited between quotation marks.

**Photos and illustrations:** Images were taken with a Canon EOS 5DS camera fitted with a Mitutoyo 10× (habitus) or 20× (genital structures) ELWD Plan Apo objective attached to a Carl Zeiss Jena Sonnar 3.5/135 MC as focus lens. Illumination was with two to three LED segments SN-1 from Stonemaster (https://www.stonemaster-onlineshop.de). Image stacks were generated using the Stackmaster macro rail (Stonemaster), and images were then assembled with the computer software Helicon Focus 4.77TM.

**Coordinates** are given in decimal notation unless cited verbatim from labels. Besides various Australian road maps, we also used Google Earth (http://earth.google.com) to locate several localities, and their coordinates are given in Degrees, Minutes (DDD° MM’). Our maps are based on “MICROSOFT ENCARTA World-Atlas 2000”.

**DNA sequencing and data analysis:** Our laboratory protocol has been explained in Hendrich et al. (2010). We used the 3’ end of the cox1 gene, widely used in diving beetle research. Each of our 27 individual vouchers bears a green cardboard label that indicates the DNA extraction number of M. Balke (e.g., “DNA M. Balke 7247”). This number links the DNA sample to the dry-mounted voucher specimen, deposited in Zoologische Staatssammlung München (**ZSM)**. We used a simple approach to calculate a neighbor-joining tree (*p*-distances) in Geneious (11.0.4.) software (Fig. 26), and subsequent visual inspection of the tree to learn whether there was any hidden diversity or haplotype sharing.

GenBank accession numbers are provided in Table 1.

**Table 1.** GenBank accession numbers for *Gibbidessus* cox1 3’end mtDNA sequences.

Species	Voucher	COI-3, accession
*Gibbidessus atomus* sp. nov.	MB 1729	FR732713
	MB 2780	FR733522
	MB 2781	FR733523
*Gibbidessus chipi*	N/A	AF484132
*Gibbidessus davidi* sp. nov.	MB 1730	FR732714
	MB 2782	FR733524
	MB 2783	FR733525
	MB 7243	MT551887
	MB 7244	MT551888
	MB 7245	MT551889
	MB 7246	MT551890
*Gibbidessus pictipes*	MB 1695	FR732684
	MB 2104	FR733521
	MB 7250	MT551896
	MB 7252	MT551895
	MB 7253	MT551894
	MB 7254	MT551893
	MB 7255	MT551892
	MB 7257	MT551891
	MB 7259	MT551900
	MB 7260	MT551899
	MB 7261	MT551898
	MB 7262	MT551897
*Gibbidessus rottnestensis* sp. nov.	MB 3921	MT551904
	MB 7247	MT551901
	MB 7248	MT551902
	MB 7249	MT551903

### Codens

**ANIC** Australian National Insect Collection, Canberra, Australia

**CFP** Collection Fernando Pederzani, Ravenna, Italy

**CGC** Collection Gilbert L. Challet, Florida, United States

**CLH** Collection Lars Hendrich, Berlin, Germany; property of the NMW

**DPAW** Department of Parks & Wildlife, Kensington, Australia

**NMW** Naturhistorisches Museum Wien, Vienna, Austria

**SAMA** South Australian Museum, Adelaide, South Australia, Australia

**WAM** Western Australian Museum, Perth, Western Australia, Australia

**ZSM** Zoologische Staatssammlung München, Munich, Germany

### Collecting procedures

Most of the specimens were collected in the flat transition zones between land and water (1 to 5 cm depth) of seasonal, mainly open swamps, smaller pools, puddles and flooded meadows, using various kinds of aquatic dip nets and plastic strainers with very fine meshes. Mesh diameters varied from 0.1 to 0.5 mm. Most specimens were collected directly from the plastic strainers with forceps and/or an aspirator. According to our knowledge and the label data studied, none of the eight species has ever been obtained via light traps.

## Taxonomy

### Checklist of *Gibbidessus* species

NSW = New South Wales; SA= South Australia; TAS, = Tasmania; VIC = Victoria; WA = Western Australia.

*G. atomus* sp. nov. south-western WA

*G. chipi* Watts, 1978 SA, VIC, TAS, NSW

*G. davidi* sp. nov. south-western WA

*G. drikdrikensis* sp. nov. south-western VIC

*G. kangarooensis* sp. nov. SA (Kangaroo Island)

*G. pederzanii* sp. nov. south-western WA

*G. pictipes* (Lea, 1899) comb. nov. south-western WA

*G. rottnestensis* sp. nov. south-western WA

#### Genus Gibbidessus

Taxon classificationAnimaliaColeopteraDytiscidae

Watts, 1978

87C0A944-8AC4-533D-AB1E-BA161FE37C77

Gibbidessus Watts, 1978: 29; gender masculine; type species: Gibbidessus chipi Watts, 1978: 52 by original designation; Biström (1988: 19); Nilsson & Hájek (2020: 108).

##### Type species.

Gibbidessus chipi Watts, 1978.

##### Diagnosis.

Very small diving beetles (1.15–1.9 mm). The smallest epigean dytiscids in Australia can be found in this genus. Body oblong-oval or elongate and fairly compactly built. Head with or without cervical line; frontally not bordered. Palpi rather slender, apically very finely bifid, in one species broad. Pronotum with a pair of basal striae. Elytron with a basal stria but without sutural striae. Punctation of elytra does not form rows. Epipleura lack a basal cavity posteriorly limited by a transverse carina. Prosternal process rather elongate, narrow, laterally distinctly marginated and with ventral surface not medially excavated. Prosternal process reaches metaventrite, which is not distinctly depressed posterior to mesocoxae. Metacoxal lines comparatively short, only slightly longer than distance between them posteriorly. Very fine punctures on either side of midline of metaventrite, not forming distinct rows. Metatrochanters and metafemora not distinctly modified (Biström 1988; Hendrich et al. 2019). Parameres symmetric. Larvae unknown.

Gibbidessus was described as a monotypic genus to accommodate G. chipi Watts, 1978. A molecular phylogenetic investigation covering all Australian Bidessini genera (Hendrich et al. 2010) recovered G. chipi, the single Uvarus species reported from Australia, Uvarus pictipes (Lea, 1899), as well as three undescribed species in one clade (Fig. 26). We refer to this clade as genus Gibbidessus. Subsequently, we assembled a multigene dataset of global Hydroporinae (Balke, work in progress), where we again recovered Gibbidessus species as well as Uvarus pictipes in one clade. The non-Australian Uvarus included in that preliminary study did form a separate, not closely related clade. Consequently, Uvarus pictipes is transferred to Gibbidessus here.

#### Gibbidessus atomus sp. nov.

Taxon classificationAnimaliaColeopteraDytiscidae

7AFC883E-9CD1-562F-8296-A38ABA622302

http://zoobank.org/713DD48F-BA41-4581-A05F-DEECAED51519

**Figures 1–4.**
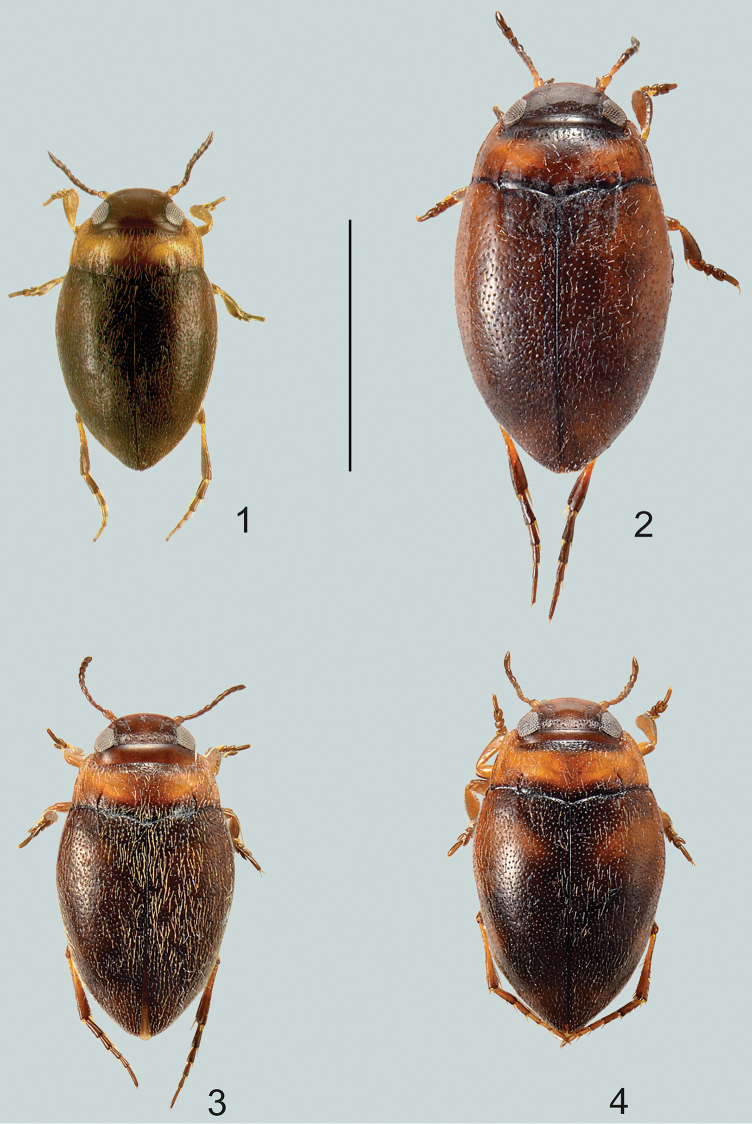
Habitus of **1***Gibbidessus atomus* sp. nov. **2***G. chipi*, **3***G. davidi*, male **4***G. davidi* female. Scale bar: 1.0 mm.

Figs 1 , 14 , 23 , 27

##### Type locality.

Western Australia, Windy Harbour Road, 11 km south of Northcliffe, small pool in sedge swamp [34°48'51S, 116°4'8E].

##### Type material.

***Holotype*,** male: “Australia: SW WA, D´Entrecasteaux NP, 11 km S, Northcliffe, 77m, 4.I.2007, 34.44.048S, 116.05.354E [34°44'0S, 116°5'13E], L. & E. Hendrich (WA 162)”, “Holotype *Gibbidessus atomus* Hendrich, Watts & Balke des. 2020” [red printed label] (WAM). ***Paratypes* (20 exs.)**. 7 specimens with same data as holotype, three specimens with “DNA M. Balke 1729”, “DNA M. Balke 2780”, “DNA M. Balke 2781” [green printed labels]; 13 exs., “AUSTRALIA/WA: D´Entrecasteaux N.P., 11 km south of Northcliffe, Windy Harbour Road, 50 m, 3.1.2000, 34°42'S, 116°05'E [34°44'0S, 116°5'13E], Hendrich leg. (loc. WA 10c/156)” (ANIC, CLH, SAMA, ZSM). All paratypes with red printed paratype labels.

##### Additional material.

1 ex., “Australia, WA, RVDLE03 Riverdale Wetland [32°59'22S, 115°47'7E], 23/09/2008, South West Catchment Council Mon.” (DPAW).

##### Diagnosis.

Very small species, externally characterised by widely rounded body, with less pronounced habitus disruption between pronotum and elytron, shiny non-microreticulate dorsal surface and vague ferruginous markings on elytra. Dorsoventrally rather domed. Cervical line present (Fig. 1).

##### Measurements.

Holotype: TL = 1.15 mm, TL-H = 1.05 mm, MW = 0.6 mm. Paratypes: TL = 1.15–1.30 mm, TL-H = 1.0–1.05 mm, MW = 0.6–0.65 mm.

***Head*:** Dark brown, around eyes almost black. Cervical line present. Strongly and coarsely punctate, rather shiny, microsculpture almost absent. Punctures weakly distributed anteriorly, strong posteriorly between eyes. Antennae relatively short, stout. Antennomeres ferruginous, darkened anteriorly.

***Pronotum*:** Ferruginous, anterior and posterior margins darker. Disc of pronotum somewhat darkened, broadest at posterior corners. Punctation very weakly, almost evenly distributed, shiny, microsculpture absent. Sides of pronotum margined and almost evenly rounded. Angle between pronotum and elytra less pronounced, basal pronotal plicae present. Striae moderately defined, on almost 1/2 length of pronotum, moderately incurved.

***Elytra*:** Dark brown with vague basal area ferruginous (Fig. 1). Coarsely and densely punctate, shiny, microsculpture absent. Striae weakly impressed, slightly straighter but shorter than basal pronotal striae.

***Ventral side*:** Ferruginous. Prothorax and apex of abdomen paler than other parts. Metacoxae and metaventrite covered with numerous larger punctures, surface shiny, without microreticulation. Abdominal ventrites with dense and finer punctures, shiny, microreticulation absent. Metacoxal lines almost straight, anteriorly slightly divergent. Epipleuron ferruginous, coarsely punctate, shiny, lacking microsculpture. Legs ferruginous with meta-/mesotibiae and meta-/mesotarsi set in black.

***Male*.** Dorsal surface with coarse punctures but otherwise with shiny surface (Fig. 1). Median lobe of aedeagus as in Fig. 14A, B. Shape of median lobe fairly uniform, bent evenly, apex straight and pointed. Paramere, as in *Limbodessus* Guignot, 1939, with hook or bent finger-like apical part with tiny setae on tip (Fig. 14C).

##### Affinities.

This species is similar to *G. davidi* sp. nov. but readily separated by its smaller size, the different colour pattern and the form of the median lobe and parameres (Figs 14, 16).

##### Etymology.

From Latin *atomus* (smallest particle), as it is the smallest epigean diving beetle in Australia described so far.

##### Distribution.

South-western Australia. Known only from the type locality in the D´Entrecasteaux National Park, south of Northcliffe and the Riverdale Wetland [32°59'22S, 115°47'7E] (Fig. 23).

##### Habitat.

Most specimens were obtained from an exposed, shallow and small roundish puddle without any vegetation, except some algae (Fig. 27B). The remaining specimens were collected in a half-shaded pool in a *Melaleuca* blackwater swamp (Fig. 27A), with few clumps of *Juncus* spp. and extensive beds of macrophytes; depth up to 20 cm; bottom of sedge-filled peat (pH 5.5), twigs and rotten leaves. The heathlands south of Northcliffe are seasonally flooded, with some permanent water bodies in the summer. At Northcliffe the species is syntopic with *G. davidi* sp. nov. (Hendrich 2001a), at the Riverdale Wetland it was collected with *G. davidi* sp. nov. and *G. rottnestensis* sp. nov.

Apart from the *Gibbidessus*, the water beetle coenosis at Northcliffe included the following species: Dytiscidae: *Limbodessus inornatus* (Sharp, 1882), *Antiporus hollingsworthi* Watts, 1997, *A. mcraeae* Watts & Pinder, 2000, *Brancuporus gottwaldi* (Hendrich, 2001), *Sternopriscus minimus* Lea, 1899, *S. eikei* Hendrich & Watts, 2007, *Exocelina ater* (Sharp, 1882); Hydrophilidae: *Enochrus eyrensis* (Blackburn, 1895), *Limnoxenus zealandicus* (Broun, 1880), *Paracymus pygmaeus* (Macleay, 1871).

#### Gibbidessus chipi

Taxon classificationAnimaliaColeopteraDytiscidae

Watts, 1978

00BCECF0-71B6-56B4-B7BE-D283858A481C

Figs 2 , 15 , 22

Gibbidessus chipi Watts, 1978: 33 (original description); Watts (1985: 24, checklist); Lawrence et al. (1987: 335, catalogue); Biström (1988: 19, systematics); Watts (2002: 31, 44, identification key, checklist); Davies et al. (2003: 24, 27, faunistics); Nilsson & Hájek (2020: 108, catalogue).

##### Type locality.

Australia, New South Wales, Collector, old farm dam [34°54'40S, 149°26'24E].

##### Type material.

***Holotype*,** male [with one paratype on the same plate]: “Collector NSW Febr 1961 C.W.”, “Holotype” [red printed label], “ANIC Database No. 25 015140” [printed label], “Holotype Gibbidessus chipi Det. C. Watts 1976 [handwritten label by Chris Watts] (ANIC). ***Paratypes* (10 exs.)**. 2 males, 4 females: “Collector NSW 2/61” [handwritten label by Chris Watts], “Paratype Gibbidessus chipi Det C. Watts 1976” [white, printed and handwritten label], “ANIC Database No 2515141” (ANIC); 2 males: “Collector NSW 2/61” [handwritten label by Chris Watts], “Paratype Gibbidessus chipi Det C. Watts 1976” [white, printed and handwritten label], “SAMA Database No 25-003994” (SAMA); 1 male, 1 female: “Dartmoor Victoria Jan 59. CW” [handwritten label], “Paratype Gibbidessus chipi Det C. Watts 1976” [white, printed and handwritten label], “SAMA Database No 25-003398” (SAMA).

##### Additional material studied

**(17 exs.):South Australia**. 2 exs., “1 km S, Nangwarry 5.X.2000, C. Watts leg.”, “SAMA Database No 25-004002”, one specimen “DNA M. Balke 2109 [green printed label] (SAMA, ZSM); 3 exs., “Fleurieu Peninsula, Myponga, A.H. Elston leg.”, “SAMA Database No 25-00399” (SAMA); 1 ex., “Mt. Crawford State Forest, Watts Gully, 3.X.1998, C. Watts leg.”, “SAMA Database No 25-003996” (SAMA). **Victoria:** 1 ex., “5.3 km S, Drik Drik,14.VIII.2004, C. Watts leg.”, “DNA Voucher d” (SAMA); 6 exs., “18 km W Casterton, 25.IX.1998, C. Watts leg.”, “SAMA Database No 25-004003” (SAMA); 3 exs., “18 km W Casterton, 25.IX.1998, C. Watts leg.” (CLH); 1 ex., “22 km W Casterton, 23.IX.1999, C. Watts leg.”, “photographed” [yellow label], “SAMA Database No 25-004004” (SAMA).

##### Diagnosis.

Medium-sized species which externally is characterised by a widely rounded body, shiny non-microreticulate dorsal surface, vague testaceous markings on elytra, and without habitus disruption between pronotum and elytron. Dorsoventrally rather domed. Cervical line present (Fig. 2).

##### Measurements.

TL = 1.5–1.55 mm, TL-H = 1.4–1.45 mm, MW = 0.86–0.98 mm.

***Head*:** Dark brown, around eyes almost black. Cervical line present. Strongly and coarsely punctate, rather shiny, microsculpture almost absent. Punctures weakly anteriorly and strongly posteriorly between eyes. Antennae relatively short, stout. Antennomeres 1-2 ferruginous, 3-11 darkened anteriorly.

***Pronotum*:** Ferruginous, anterior and posterior margins darker. Disc of pronotum somewhat darkened, broadest at posterior corners. Punctation very weakly punctate almost evenly distributed, shiny and microsculpture absent. Sides of pronotum margined and almost evenly rounded. Angle between pronotum and elytra not pronounced, basal pronotal plicae present. Striae well defined, almost 1/2 length of pronotum, strongly incurved.

***Elytra*:** Ferruginous, with vague areas darkened (Fig. 2). Coarsely and densely punctate, shiny, microsculpture absent. Striae strongly impressed, same length as basal pronotal striae but slightly straighter.

***Ventral side*:** Ferruginous. Prothorax and abdomen paler than other parts. Metacoxae and metaventrite covered with numerous larger punctures, surface shiny, without microreticulation. Abdominal ventrites with dense and fine punctures, shiny, microreticulation absent. Metacoxal lines almost straight, anteriorly slightly divergent. Epipleuron testaceous, with few coarse punctures, shiny, lacking microsculpture. Legs ferruginous with meta-/mesotibia and meta-/mesotarsi set in black.

***Male***. Dorsal surface with coarse punctures but otherwise with shiny surface Fig. 2. Median lobe of aedeagus as in Fig. 15A, B. Shape of median lobe fairly uniform, apex in lateral view straight and pointed, in ventral view very broad and rounded at apex. Parameres bi-segmented and elongated with few setae at apex (Fig. 15C).

##### Affinities.

This species is similar to *G. drikdrikensis* sp. nov. but readily separated by its smaller size, and the form of the median lobe (Figs 15, 17).

##### Distribution.

South-eastern Australia, from the Lofty Ranges near Adelaide and north-eastern Tasmania to Canberra (Watts 1978). Also recorded from King Island, Bertie Lagoon [39°42'36S, 144°4'24E] (Davies et al. 2003), northwest of Tasmania (Fig. 22).

##### Habitat.

The type specimens were collected in an old farm dam and its flood zone, overgrown by rich vegetation. In Victoria and South Australia most of the specimens were collected in small shallow pools and seasonal wetlands. A single specimen from South Australia (Mount Crawford State Forest, Watts Gully) has been found in a shallow, slow flowing temporary forest creek. At Dri Drik, in Victoria, the species is syntopic with *G. drikdrikensis* sp. nov.

#### Gibbidessus davidi sp. nov.

Taxon classificationAnimaliaColeopteraDytiscidae

91450849-DDF9-5F46-B94A-9DF53F701C3E

http://zoobank.org/4D235E7C-F517-4C1D-A3C7-0C87BDA34515

Figs 3 , 4 , 12 , 16 , 23 , 27 , 28

##### Type locality.

Western Australia, Perth, suburb Success, Beeliar Regional Park, shallow peaty puddle [32°8'4S, 115°50'22E].

##### Type material.

***Holotype*,** male: “Australia, WA, Perth, Success, Beeliar RP, shallow peaty puddle 32°8'4.97"S, 115°50'22.78"E 21.-31.10.2015 L. Hendrich (WA 1/15)”, “Holotype *Gibbidessus davidi* Hendrich, Watts & Balke des. 2020” (WAM) [red printed label]. ***Paratypes* (370 exs.)**. 354 specimens with same data as holotype (ANIC, CGC, CLH, SAMA, WAM, ZSM); 4 exs, “Australia: SW WA, D´Entrecasteaux NP, 11 km S, Northcliffe, 77m, 4.I.2007, 34.44.048S, 116.05.354E [34°44'0S, 116°5'13E], L. & E. Hendrich (WA 162)” (ZSM); 10 exs., “Australia, WA, Albany Hwy, Muir Lakes Nature Reserve, SW part of Byenup Lagoon, 4.& 5.1.2000, 34°30'4S, 116°44'19E, Hendrich leg. (loc. WA 11/157)” (CLH, ZSM); 1 ex., “Australia, WA, Barlee Brook Dickson Road (A) [34°12'17S, 115°46'18E], DON03, 21/10/2005, South West Forest Monitoring” (DPAW); 1 ex., “Australia, WA, Fish Creek (A) [34°37'29S, 116°26'11E], SHA22, 17/10/2010, South West Forest Monitoring” (DPAW); 4 exs., “Australia, WA, RVDLE03 Riverdale Wetland [32°59'23S, 115°47'23E], 23/09/2008, South West Catchment Council Mon.” (DPAW). All paratypes with red printed paratype labels.

##### Diagnosis.

Small species which externally is characterised by a wide rounded body, shiny non-microreticulate dorsal surface, vague testaceous markings on elytra, and without habitus disruption between pronotum and elytron. Dorsoventrally rather domed. Cervical line present (Fig. 3).

##### Measurements.

Holotype: TL = 1.45 mm, TL-H = 1.35 mm, MW = 0.83 mm. Paratypes: TL = 1.35–1.5 mm, TL-H = 1.15–1.4 mm, MW = 0.8–0.9 mm.

***Head*:** Ferruginous, around eyes almost black. Cervical line present (Fig. 12A). Strongly and coarsely punctate, rather shiny, microreticulation present. Punctures weak anteriorly and strongly posteriorly between eyes. Antennae relatively short, stout. Antennomeres 1-8 ferruginous, 9-11 darkened anteriorly.

***Pronotum*:** Ferruginous, anterior and posterior margins darker. Disc of pronotum somewhat darkened, broadest at posterior corners. Punctation of pronotum very weak, almost evenly distributed, shiny and microsculpture absent. Sides of pronotum margined and almost evenly rounded. Angle between pronotum and elytra less pronounced, basal pronotal plicae present. Striae moderately defined, almost 1/2 length of pronotum, strongly incurved.

***Elytra*:** Dark brown with vague basolateral area ferruginous (Fig. 3). Coarsely and densely punctate, shiny, microsculpture absent. Striae deeply impressed, straight but shorter than basal pronotal striae.

***Ventral side*:** Ferruginous. Prothorax and abdomen paler than other parts. Metacoxae and metaventrite covered with larger punctures, surface shiny, without microreticulation. Abdominal ventrites with dense and finer punctures, shiny, microreticulation absent. Metacoxal lines almost straight, anteriorly not divergent. Epipleuron ferruginous, with few coarse punctures, shiny, lacking microsculpture. Legs ferruginous with meta-/mesotibia and meta-/mesotarsi set in black.

***Male*.** Smaller and more elongate than female (Fig. 4). Median lobe of aedeagus as in Fig. 16A, B. Shape of median lobe in lateral view, straight and fairly uniform, in ventral view broad, with a thorn on each side, and rounded at apex. Parameres bi-segmented and elongated with few setae at apex (Fig. 16C, D).

##### Affinities.

This species is similar to *G. atomus* sp. nov. but readily separated by its larger size, the different colour pattern and the form of the median lobe and parameres (Figs 14, 16). From *G. pederzanii* sp. nov. it can be distinguished by the less roundish body and the form of the median lobe and parameres (Figs 16, 19).

##### Etymology.

The beetle is named after the son of the first author, David Hendrich. The specific epithet is a substantive in the genitive case.

##### Distribution.

South-western Australia. From Perth in the north to D´Entrecasteaux National Park in the south (Fig. 23).

##### Habitat.

In the Northcliffe area most specimens were obtained from an exposed, shallow and small roundish puddle, without any vegetation, except some algae. The other specimens were collected in a half-shaded pool in a Melaleuca blackwater swamp, with few clumps of *Juncus* spp. and extensive beds of macrophytes; depth up to 20 cm; bottom consisted of sedge-filled peat (pH 5.5), twigs and rotten leaves (Figs 27, 28). The whole area south of Northcliffe is seasonally flooded with some permanent central water bodies in summer. In the D´Entrecasteaux NP the species is syntopic with *G. atomus* sp. nov., and around Perth in the Beeliar Regional Park with *G. rottnestensis* sp. nov. At the Riverdale Wetland Reserve *G. davidi* sp. nov. was syntopic with *G. atomus* sp. nov. and *G. rottnestensis* sp. nov.

Apart from the *Gibbidessus*, the water beetle coenosis at Northcliffe included the following species: Dytiscidae: *Limbodessus inornatus*, *Antiporus hollingsworthi*, *A. mcraeae*, *Brancuporus gottwaldi*, *Sternopriscus minimus*, *S. eikei*, *Exocelina ater*; Hydrophilidae: *Enochrus eyrensis*, *Limnoxenus zealandicus*, *Paracymus pygmaeus* (see Hendrich 2001a). In the Beeliar Park the two *Gibbidessus* species share their habitat with *Limbodessus inornatus*, *Paroster insculptilis* (Clark, 1862), *Exocelina ater*, *Rhantus suturalis* (Macleay, 1825) and *Rhantus simulans* Régimbart, 1908.

#### Gibbidessus drikdrikensis sp. nov.

Taxon classificationAnimaliaColeopteraDytiscidae

C7A6160C-AA5C-56E0-9A0C-94F7EAF03F48

http://zoobank.org/281FB8F4-C366-40CA-9358-6BF75CBCE8DD

Figs 5 , 17 , 22

##### Type locality.

Australia, Victoria, Drik Drik, old farm dam [37°59'27S, 141°17'18E].

##### Type material.

***Holotype*:** Male, “1 km S, Drik Drik Vic. 24/9/98 C. Watts”, “Photographed”, “SAMA Database No 25-004000”, “Holotype *Gibbidessus drikdrikensis* sp.nov. Hendrich, Watts & Balke des. 2020” [red printed label] (SAMA). ***Paratypes* (6 exs.):** 6 specimens with same data as holotype. Two specimens with “DNA voucher b” and “DNA voucher c” and one with a yellow prointed label “photographed” (CLH, SAMA). All paratypes are provided with printed red paratype labels.

##### Diagnosis.

**Figures 5–7.**
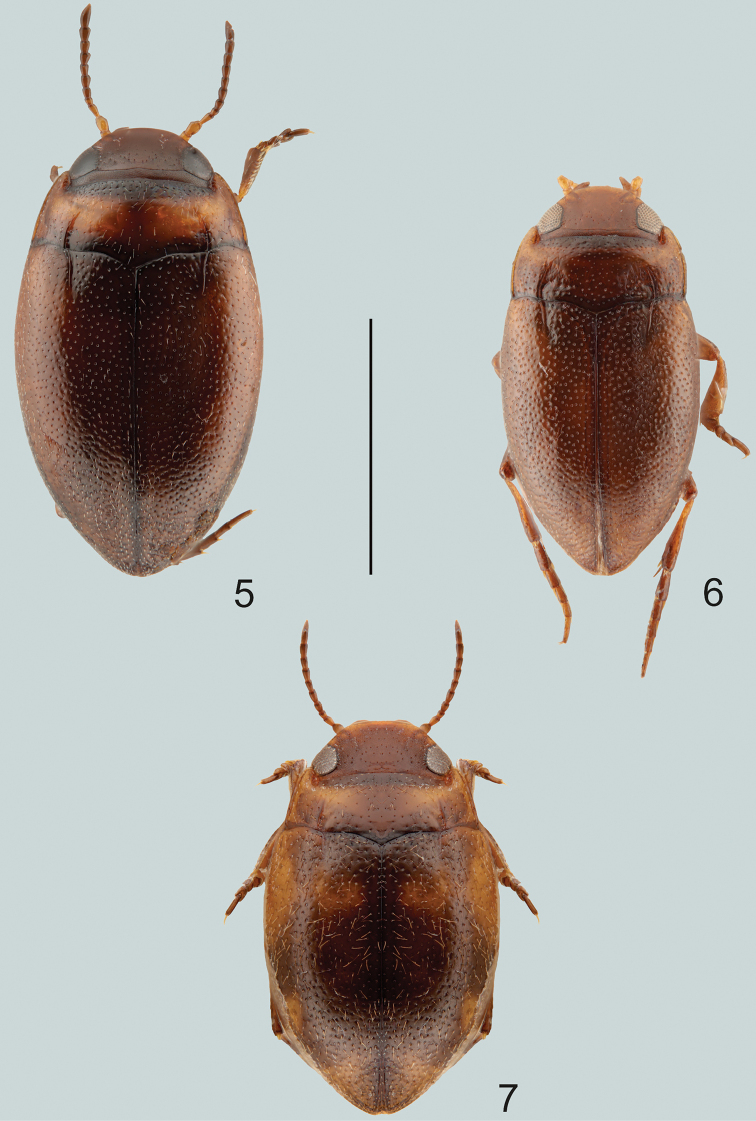
Habitus of **5***Gibbidessus drikdrikensis* sp. nov. **6***G. kangarooensis* sp. nov. **7***G. pederzanii* sp. nov. Scale bar: 1.0 mm.

Medium-sized species which externally is characterised by a wide rounded body, shiny non-microreticulate dorsal surface, vague testaceous markings on elytra, and without slight habitus disruption between pronotum and elytron. Dorsoventrally rather domed. Cervical line present (Fig. 5).

##### Measurements.

Holotype: TL = 1.7 mm, TL-H = 1.5 mm, MW = 0.98 mm. Paratypes: TL = 1.6–1.7 mm, TL-H = 1.4–1.5 mm, MW = 0.86–0.98 mm.

***Head*:** Dark brown, around eyes almost black. Cervical line present. Strongly and coarsely punctate, rather shiny, microsculpture almost absent. Punctures weakly anteriorly and strongly posteriorly between eyes. Antennae relatively short, stout. Antennomeres 1 and 2 ferruginous, 3-11 darkened anteriorly.

***Pronotum*:** Ferruginous, anterior and posterior margins darker. Disc of pronotum somewhat darkened, broadest at posterior corners. Punctation very weak, almost evenly distributed, shiny and microsculpture absent. Sides of pronotum margined and almost evenly rounded. Angle between pronotum and elytra not pronounced, basal pronotal plicae present. Striae well defined, almost 1/2 length of pronotum, strongly incurved.

***Elytra*:** Ferruginous, with vague areas darkened (Fig. 5). Coarsely and densely punctate, shiny, microsculpture absent. Striae strongly impressed, same length as basal pronotal striae but slightly straighter.

***Ventral side*:** Ferruginous. Prothorax and abdomen paler than other parts. Metacoxae and metaventrite covered with numerous larger punctures, surface shiny, without microreticulation. Abdominal ventrites with dense and finer punctures, shiny, microreticulation absent. Metacoxal lines almost straight, anteriorly slightly divergent. Epipleuron testaceous, with few coarse punctures, shiny, lacking microsculpture. Legs ferruginous meta-/mesotarsi set in black.

***Male*.** Dorsal surface with coarse punctures but otherwise with shiny surface (Fig. 5). Median lobe of aedeagus as in Fig. 17A, B. Shape of median lobe fairly uniform, evenly bent in lateral view, apex straight and pointed at tip in ventral view. Parameres bi-segmented, broad and with few setae at apex (Fig. 17C, D).

##### Affinities.

This species is very similar to *G. chipi* but readily separated by its larger size and the form of the median lobe and parameres (Figs 15, 17).

##### Etymology.

The species is named after the type locality. The specific epithet is a substantive in the genitive case.

##### Distribution.

Only known from the type locality Drik Drik in south-western Victoria (Fig. 22).

##### Habitat.

The few specimens were collected in shallow water at the edge of a large, exposed but shallow farm dam, overgrown with grasses and sedges. The species is syntopic with *G. chipi*.

#### Gibbidessus kangarooensis sp. nov.

Taxon classificationAnimaliaColeopteraDytiscidae

C8AEA995-DFE8-5411-9032-FA486A772502

http://zoobank.org/9CD26871-28DA-4A6E-A9FC-DBC0F74A6DC6

Figs 6 , 18 , 22

##### Type locality.

South Australia, Kangaroo Island, Eleaner River at South Coast [Road] Crossing, edge sample [35°56'S, 137°14'E].

##### Type material.

***Holotype*:** Male, “Eleaner R. S, Coast rd AWQ [Australian Water Quality] survey 8/11/95 site 3714 [35°56'S, 137°14'E]” “Holotype *Gibbidessus kangarooensis* sp. nov. Hendrich, Watts & Balke des. 2020” [red printed label] (SAMA).

##### Diagnosis.

Small species which externally is characterised by a more elongate body, shiny non-microreticulate dorsal surface, and with well pronounced habitus disruption between pronotum and elytron. Dorsoventrally rather flattened. Without cervical line but rather a few punctures instead (Fig. 6).

##### Measurements.

Holotype: TL = 1.55 mm, TL-H = 1.4 mm, MW = 0.88 mm.

***Head*:** Ferruginous, without cervical line but rather a few punctures instead. Evenly and coarsely punctate, shiny, microsculpture absent. Punctures weakly anteriorly and strongly posteriorly between eyes. Antennae missing.

***Pronotum*:** Ferruginous, anterior and posterior margins darker, broadest at middle. Punctation weak anteriorly but quite strong on posterior half and on lateral sides, almost evenly distributed, shiny and microsculpture absent. Sides of pronotum broadly margined and almost evenly rounded. Angle between pronotum and elytra well pronounced, basal pronotal and elytral plicae present. Striae moderately defined, almost 1/2 length of pronotum, strongly incurved.

***Elytra*:** Dark brown with vague basal area ferruginous (Fig. 6). Coarsely and densely punctate, shiny, microsculpture absent. Striae weakly impressed, slightly straighter and of same length as basal pronotal striae.

***Ventral side*:** Ferruginous. Prothorax and apex of abdomen paler than other parts. Metacoxae and metaventrite covered with numerous larger punctures, surface shiny, without microreticulation. Abdominal ventrites with finer punctures, shiny, microreticulation absent. Metacoxal lines almost straight, anteriorly slightly divergent. Epipleuron ferruginous, coarsely punctate, shiny, lacking microsculpture. Legs ferruginous with meta-/mesotibia and meta-/mesotarsi somewhat darkened.

***Male*.** Dorsal surface with coarse punctures but otherwise with shiny surface (Fig. 6). Median lobe of aedeagus as in Fig. 18A, B. Shape of median lobe, bent evenly and fairly uniform in lateral view, in ventral view pointed at apex. Parameres bi-segmented, elongated, and with few setae at apex (Fig. 18C, D).

***Female*.** Unknown.

##### Affinities.

This species is similar to *G. pictipes* but readily separated by the different colour pattern and the more flattened body. Furthermore, both species can be separated by the form of the median lobe and parameres (Figs 18, 20).

##### Etymology.

The species is named after the type locality. The specific epithet is a substantive in the genitive case.

##### Distribution.

A rare species, only known from the type locality on Kangaroo Island, South Australia (Fig. 22).

##### Habitat.

The single specimen was collected at the edge of the Eleaner River in the southern part of Kangaroo Island. Most probably this is not the original habitat of the species. Almost all *Gibbidessus* inhabit more seasonal, open wetlands, overgrown with sedges and rushes.

#### Gibbidessus pederzanii sp. nov.

Taxon classificationAnimaliaColeopteraDytiscidae

E328E66D-BD01-54AD-AD8F-D4978F9E5E0F

http://zoobank.org/9389E4E1-D5E3-4063-A0F3-33C7FAFD045E

Figs 7 , 12 , 19 , 25

##### Type locality.

Australia, Western Australia, creek around Nannup [33°58'S, 115°45'E].

##### Type material.

***Holotype*:** Male, “Australia (WA) Nannup env. roadside creeks 1/12/98 Pederzani”, “Holotype *Gibbidessus pederzanii* Hendrich, Watts & Balke des. 2020” [red printed label] (SAMA). ***Paratypes* (13 exs.):** All specimens with same data as holotype. Two specimens with “SAMA Database No 25-001593” and one with a yellow printed label “photographed” (CFP, CLH, SAMA, ZSM). All paratypes are provided with printed red paratype labels.

##### Diagnosis.

Medium-sized species which externally is characterised by a rounded habitus, without disruption between pronotum and elytron, and shiny, non-microreticulate dorsal surface with testaceous markings on elytra. Dorsoventrally rather arched. Without cervical line but rather a few punctures instead (Fig. 7).

##### Measurements.

Holotype: TL = 1.5 mm, TL-H = 1.3 mm, MW = 0.85 mm. Paratypes: TL = 1.5–1.6 mm, TL-H = 1.3–1.4 mm, MW = 0.85–0.95 mm.

***Head*:** Ferruginous, around eyes almost black, without cervical line but rather a few punctures instead (Fig. 12B). Coarsely punctate, rather shiny, weak microreticulation visible. Punctures weakly anteriorly and strongly posteriorly between eyes. Antennae relatively short, stout. Antennomeres 1 and 2 ferruginous, 3-11 darkened anteriorly.

***Pronotum*:** Ferruginous, anterior and posterior margins, between striae, slightly darker, broadest at posterior corners. Punctation very weak almost evenly distributed, shiny and microsculpture absent. Sides of pronotum margined and almost evenly rounded. Angle between pronotum and elytra less pronounced, basal pronotal plicae present. Striae moderately defined, almost 1/2 length of pronotum, slightly incurved.

***Elytra*:** Dark brown with vague basolateral and apical area ferruginous (Fig. 7). Coarsely and densely punctate, shiny, microsculpture absent. Striae deeply impressed, straight but shorter than basal pronotal striae.

***Ventral side*:** Ferruginous. Prothorax and abdomen paler than other parts. Metacoxae and metaventrite covered with larger punctures, surface shiny, without microreticulation. Abdominal ventrites with dense and finer punctures, shiny, microreticulation absent. Metacoxal lines almost straight, anteriorly not divergent. Epipleuron testaceous, with few coarse punctures, shiny, lacking microsculpture. Legs ferruginous with meta- and mesotarsi somewhat darkened.

***Male*.** Dorsal surface with coarse punctures but otherwise with shiny surface (Fig. 7). Median lobe of aedeagus as in Fig. 19A, B. Shape of median lobe, almost straight and fairly uniform in lateral view, in ventral view rounded at apex. Parameres bi-segmented, elongated, and without setae at apex (Fig. 19C, D).

##### Affinities.

This species is similar to *G. davidi* sp. nov. but readily separated by the different colour pattern, the more roundish body (Figs 4, 7), the larger punctation on elytra, and the form of the median lobe and parameres (Figs 16, 19).

##### Etymology.

The species is named after our colleague, the dytiscid specialist Fernando Pederzani (Ravenna, Italy), who collected the type material. The specific epithet is a substantive in the genitive case.

##### Distribution.

South-western Australia. A rare species, which is only known from the type locality somewhere around Nannup in south-western Australia. Most probably a more inland species and restricted to forested areas and not in heathland or coastal sedge swamps (Fig. 25).

##### Habitat.

All specimens were collected in shallow water at the edge of a small slow flowing forest creek (F. Pederzani in litt.).

#### Gibbidessus pictipes

Taxon classificationAnimaliaColeopteraDytiscidae

(Lea, 1899) comb. nov.

D81AB87E-52C7-5080-ACCE-2FF14EE8C4C8

**Figures 8–11.**
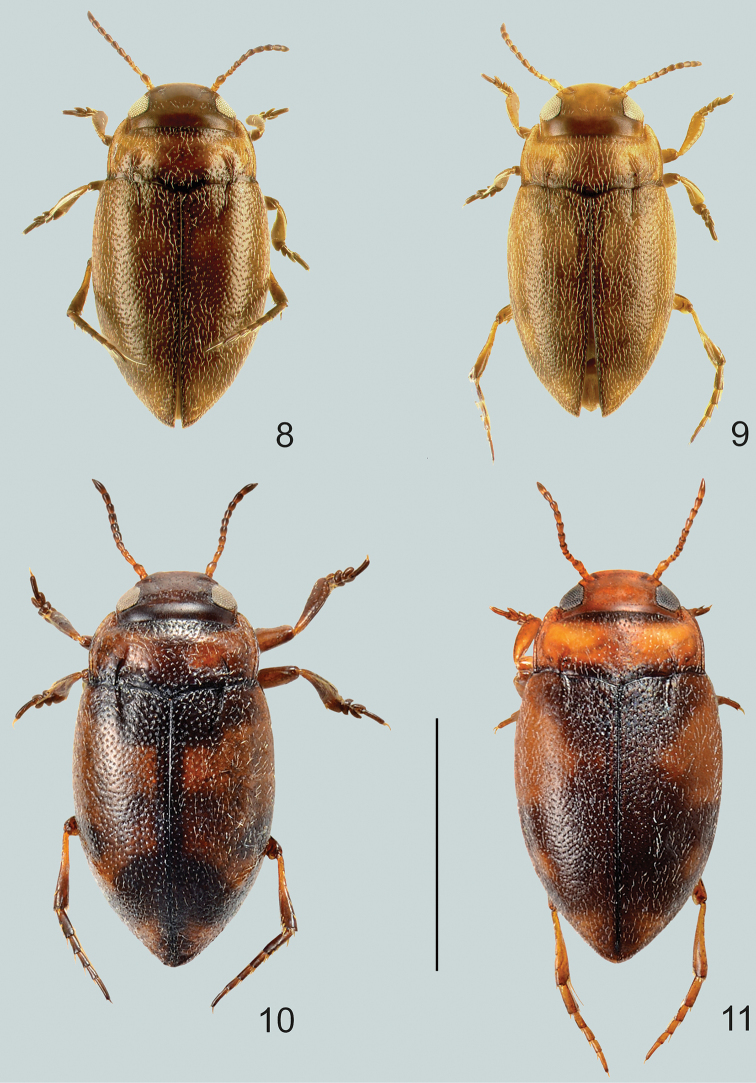
Habitus of **8***G. pictipes*, male **9***G. pictipes*, female **10***G. rottnestensis* sp. nov., male **11***G. rottnestensis* sp. nov., female. Scale bar: 1.0 mm.

Figs 8 , 9 , 13 , 20 , 30 , 31

Bidessus pictipes Lea, 1899: 523 (original description).
Uvarus pictipes (Lea, 1899): Watts (1978: 33, comb. nov., redescription); Watts (1985: 24, checklist); Lawrence et al. (1987: 335, catalogue); Biström (1988: 10, systematics); Hendrich (2001a: 302, faunistics, habitat); Hendrich (2001b: 21, faunistics, habitat); Watts (2002: 31, 44, identification key, checklist); Nilsson & Hájek (2020: 128, catalogue).

##### Type locality.

Australia, south-western Australia, Pinjarrah [32°37'56S, 115°51'49E].

##### Type material.

***Syntype*,** female, “pictipes Lea Type Pinjarrah” (handwritten label), “Bidessus pictipes Lea W. Australia TYPE” (handwritten label), “SAMA Database No 25-001599” (SAMA).

##### Additional material studied

**(320 exs.)**. 51 exs., “WA Lake Nalyerin 33 08S, 116 22E CHS, Watts 6/10/03”, “SAMA Database 25-009282” (SAMA); 4 exs., “Nalyeen Lake [Nalyerin] WA J. McRae 8/10/97”, “SAMA Database No 25-002918” (SAMA); 1 ex., “WA Byenup Lagoon NR 21/9/00 C.H.S. Watts” “SAMA Database No 25-002922” (SAMA). 2 exs., “Australia, WA, SWA31 (A), Helena River, 22/08/2005, South-west Forest Monitoring” (DPAW); 1 ex., “Australia, WA, HAR21 (A), Stirling Dam 2, 7/09/2006, South-west Forest Monitoring” (DPAW); 3 exs., “Australia, WA, SPM011 (B) Kulicup Swamp, 6/11/1998, Salinity Action Plan Wetland Monitoring Programme” (DPAW); 1 ex., “Australia, WA, SPM011 (A) Kulicup Swamp [34°20'1S, 116°47'17E], 23/10/2002, Salinity Action Plan Wetland Monitoring Programme” (DPAW); 1 ex., “Australia, WA, SPM011 (B) Kulicup Swamp [34°20'1S, 116°47'17E], 23/10/2002, Salinity Action Plan Wetland Monitoring Programme” (DPAW); 2 exs., “Australia, WA, SPM011 (A) Kulicup Swamp [34°20'1S, 116°47'17E], 11/10/2000, Salinity Action Plan Wetland Monitoring Programme” (DPAW); 2 exs., “Australia, WA, SPM011 (B) Kulicup Swamp [34°20'1S, 116°47'17E], 11/10/2000, Salinity Action Plan Wetland Monitoring Programme” (DPAW); 1 ex., “Australia, WA, SPM024 (A) Lake Pleasant View [34°49'S, 118°10'E], 24/10/1999, Salinity Action Plan Wetland Monitoring Programme” (DPAW); 3 exs., “Australia, WA, SPM024 (A) Lake Pleasant View [34°49'S, 118°10'E], 24/10/2003, Salinity Action Plan Wetland Monitoring Programme” (DPAW); 2 ex., “Australia, WA, SPM024 (B) Lake Pleasant View [34°49'S, 118°10'E], 24/10/2001, Salinity Action Plan Wetland Monitoring Programme” (DPAW); 4 exs., “Australia, WA, SPM024 (A) Lake Pleasant View [34°49'S, 118°10'E], 24/10/1999, Salinity Action Plan Wetland Monitoring Programme” (DPAW); 1 ex., “Australia, WA, RUAB01 (A) Ruabon Road 01 [33°37'S, 115°27'E], 11/10/2007, South-west Catchment Council Wetland monitoring” (DPAW); 1 ex., “Australia, WA, RVDLE03 (A) Riverdale 03 [32°59S, 115°47E], 4/10/2007, South-west Catchment Council Wetland monitoring” (DPAW); 30 exs., “Australia, WA, SPS111 (A), Boyacup Bridge Swamp [34°13'S, 117°15'E], 10/10/1998, Salinity Action Plan Wetland Biological Survey” (DPAW); 1 ex., “Australia, WA, SPS032 (A), Qualeup Lake [33°50'S, 116°45'E], 9/10/1998, Salinity Action Plan Wetland Biological Survey” (DPAW); 2 exs., “Australia, WA, SPS103 (A), Lake Poorginup [34°32'S, 116°44'E], 2/10/1998, Salinity Action Plan Wetland Biological Survey” (DPAW); 4 exs., “Australia, WA, SPS104 (A), Pindicup Lake [34°25'S, 116°43'E], 2/10/1998, Salinity Action Plan Wetland Biological Survey”, one specimen “M. Balke DNA 7250” [green printed label] (DPAW, ZSM); 1 ex., “Australia, WA, MUB030 (A), Pindicup Lake [34°25'S, 116°43'E], 25.09.2014, Muir-Byenup Survey” (DPAW); 11 exs., “Australia, WA, MUB030 (A), Pindicup Lake, 25.09.2014, Muir-Byenup Survey (DPAW); 4 exs., “Australia, WA, SPS105 (A) Kodjinup Melaleuca Swamp [34°23'S, 116°39'E], 2/10/1998, Salinity Action Plan Wetland Biological Survey” (DPAW); 30 exs, “Australia, WA, SPS108 (A) Pillenorup Swamp, 30/09/1998, Salinity Action Plan Wetland Biological Survey” (DPAW); 7 exs., “Australia, WA, SPS113 (A) Tucker’s Road Melaleuca Swamp, 27/08/1998, Salinity Action Plan Wetland Biological Survey” (DPAW); 1 ex., “Australia, WA, LVR003 (A) Lower Vasse River Site 3, 4/10/2001, Lower Vasse River Clean-up Program” (DPAW); 1 ex., “Australia, WA, LVR004 (A), Lower Vasse River Site 4, 5/10/2001, Lower Vasse River Clean-up Program” (DPAW); 1 ex., “Australia, WA, LVR005 (A), Lower Vasse River Site 5, 5/10/2001, Lower Vasse River Clean-up Program” (DPAW); 6 exs., “Australia, WA, JCS019 (A), Sedge Swamp W of Deadhorse Soak [29°54'7S, 115°1'11E], 21/09/2011, Jurien Coastal Survey” (DPAW); 1 ex., “Australia, WA, Mulgarnup MUB 012, 28.01.2004, Andrew Storey leg.” (DPAW); 2 exs., “Australia, WA, Mulgarnup MUB 012, 1.10.2014, M. Pennifold leg.”, two specimens “M. Balke DNA 7252”, “M. Balke DNA 7253” [green printed labels] (ZSM); 4 exs., “Australia, WA, W Kodjinup #18 [34°23'S, 116°39'E], 28.01.2004, Andrew Storey leg.” (DPAW); 1 ex., “Australia, WA, Wimbalup #52 [34°29'S, 116°50'E], 29.11.2003, Andrew Storey leg.” (DPAW); 11 exs., “Australia, WA, MUB019 (A), Galamup Swamp [34°26'S, 116°45'E], 30.09.2014, Muir-Byenup Survey”, one specimen “M. Balke DNA 7262” [green printed label] (DPAW, ZSM); 23 exs., “Australia, WA, MUB035, NE Unicup NR [34°20'S, 116°43'E], 24.09.2014, Muir-Byenup Survey”, one specimen “M. Balke DNA 7257” [green printed label] (DPAW, ZSM); 43 exs., “Australia, WA, MUB037 (A) S, Kulunilup NR [34°20'S, 116°47'E], 25.09.2014, Muir-Byenup Survey”, three specimens “M. Balke DNA 7259”, “M. Balke DNA 7260”, “M. Balke DNA 7261” [green printed labels] (DPAW, ZSM); 11 exs., “Australia, WA, MUB011 (A) Kulunilup Lake [34°20'S, 116°47'E], 25.09.2014, Muir-Byenup Survey” (DPAW); 2 exs., “Australia, WA, MUB008 (B) Noobijup Swamp [34°23'S, 116°47'E], 24.09.2014, Muir-Byenup Survey” two specimens “M.Balke DNA 7254”, “M. Balke DNA 7255” [green printed labels] (DPAW, ZSM); 1 ex., “Australia, WA, MUB005 (A), Yarnup Swamp [34°22'26S, 116°52'4E], 22.09.2014, Muir-Byenup Survey” (DPAW); 12 exs., “Australia, WA, MUB012 (A), Mulgarnup Swamp [34°15'1S, 116°41'44E], 01.10.2014, Muir-Byenup Survey” (DPAW); 1 ex., “Australia, WA, Twin Swamps NW [31°43'7S, 116°0'50E], 24/09/1992, Twin Swamps/Ellen Brook Survey” (DPAW); 1 ex., “Australia, WA, SPS031, 08/10/1997, SAP Survey” (DPAW); 1 ex., “Australia, WA, ABP041, 14/09/2007, Avon Baselining Project” (DPAW); 26 exs., “Australia, WA, Albany Hwy, Muir Lakes Nature Reserve, SW part of Byenup Lagoon, 4.& 5.1.2000, 34°29'S, 116°44'E, Hendrich leg. (loc. WA 11/157)” (CLH, ZSM); 18 ex., “Australia, WA, Albany, 3 km ENE Manypeaks, Lake Pleasant Nature Reserve, 7.1.2000, 34°49'S, 118°10'E, Hendrich leg. (loc. WA 13/159) (CLH, ZSM); 38 exs., “Australia, WA, Darling Range, Lane Poole Conservation Reserve, Nalyerin Lake, 300 m, 29. & 30.12.1999, 33°8.51'S, 116°22.15'E, Hendrich leg. (loc. WA 4/151)” (CGC, CLH, ZSM); 1 ex., “Australia: SW WA, 3 km NE Manypeaks, Lake Pleasant View NR, 91m, 2.I.2007, 34°49'S, 118°10'E, L. & E. Hendrich leg. (WA 160)” (ZSM).

##### Note.

Watts (1978), who moved *Bidessus pictipes* to *Uvarus*, already noticed that this species might not belong to *Uvarus*: “It is with considerable hesitation that I place the following species in this genus. I suspect that it will eventually prove to belong to a genus of its own”.

##### Diagnosis.

Small and dark brown species, with vague testaceous markings on elytra, and with habitus disruption between pronotum and elytron. Dorsoventrally rather flattened. Head without cervical line but rather a few punctures instead (punctures not obvious in females) (Fig. 8).

***Measurements*:** Lectotype, female: TL = 1.45 mm, TL-H = 1.25 mm, MW = 0.82 mm. Additional material: TL = 1.45–1.6 mm, TL-H = 1.3–1.4 mm, MW = 0.8–0.83 mm.

**Figure 12.**
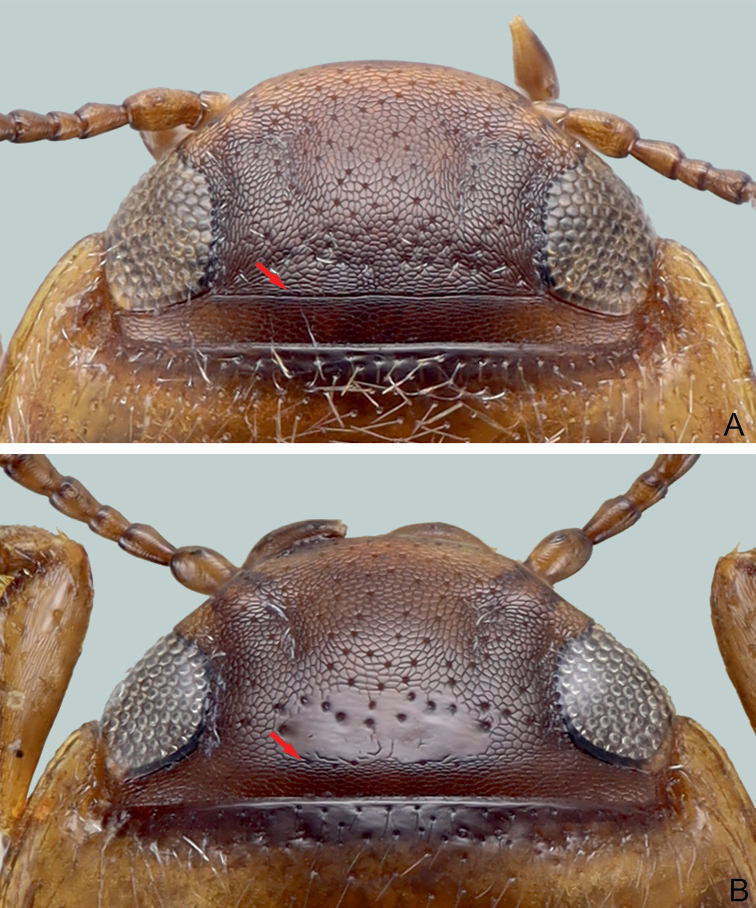
Head of *G. davidi* sp. nov. **A** with cervical line (red arrow), and head of *G. pederzanii* sp. nov. **B** without cervical line but rather a few punctures instead (red arrow).

**Figure 13.**
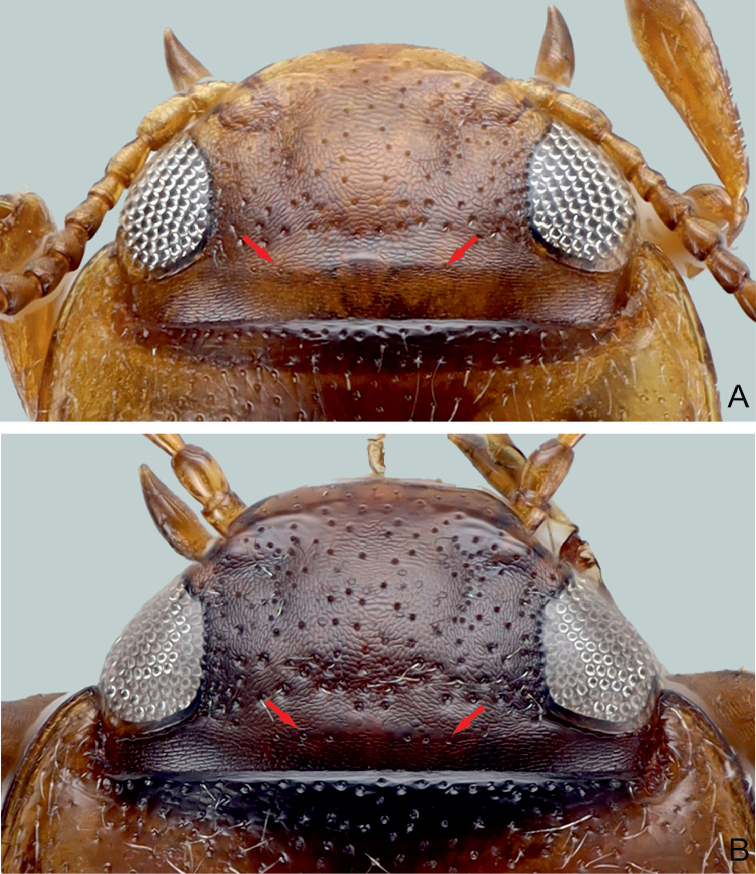
Head of *G. pictipes***A** without cervical line (red arrows), and of *G. rottnestensis* sp. nov. **B** without cervical line but rather a few punctures instead (red arrows).

***Head*:** Dark brown to ferruginous, without cervical line but rather a few punctures instead (punctures not obvious in females) (Fig. 13A). Evenly and coarsely punctate, shiny but with weak microreticulation. Punctures weakly anteriorly and strongly posteriorly between eyes. Antennae relatively short, stout. Antennomeres 1–3 ferruginous, 4–11 darkened anteriorly.

***Pronotum*:** Ferruginous, anterior and posterior margins darker, broadest at middle. Punctation very weak, almost evenly distributed, shiny and microsculpture absent. Sides of pronotum margined and almost evenly rounded. Angle between pronotum and elytra well pronounced, basal pronotal plicae present. Striae moderately defined, almost 1/2 length of pronotum, strongly incurved.

**Figures 14, 15.**
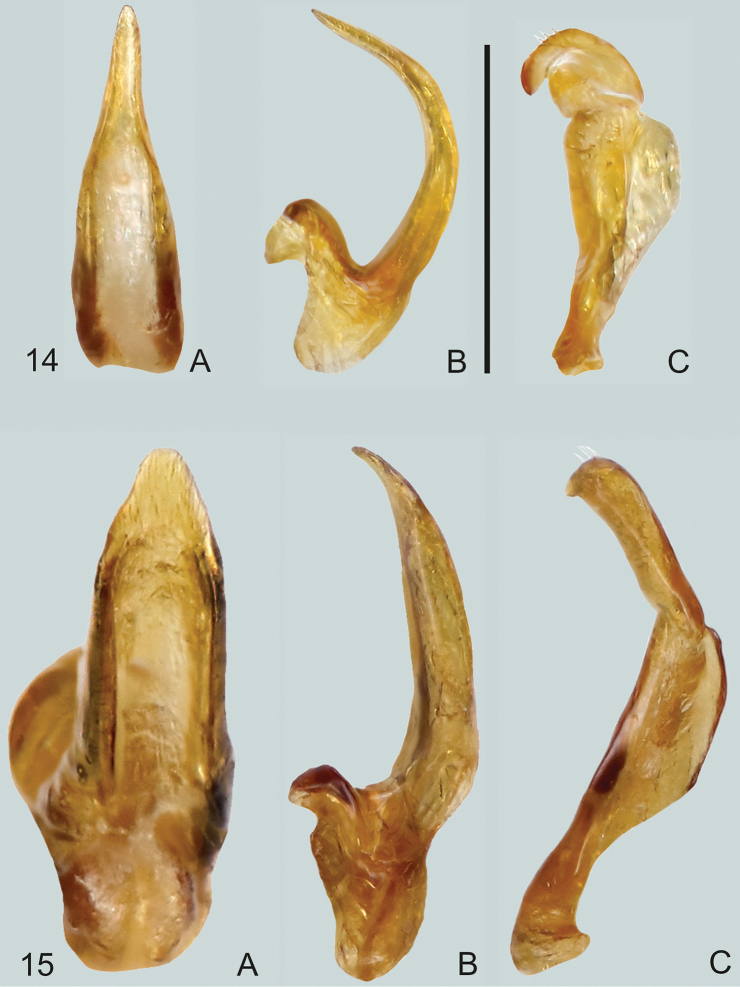
**14***Gibbidessus atomus* sp. nov. **15***G. chipi***A** median lobe in ventral view **B** median lobe in lateral view, left side **C** left paramere in lateral view. Scale bar: 0.2 mm.

***Elytra*:** Dark brown with vague basal area ferruginous (Fig. 8). Coarsely and densely punctate, shiny, microsculpture absent. Striae weakly impressed, slightly straighter than in female specimens and of same length as basal pronotal striae.

**Figures 16, 17.**
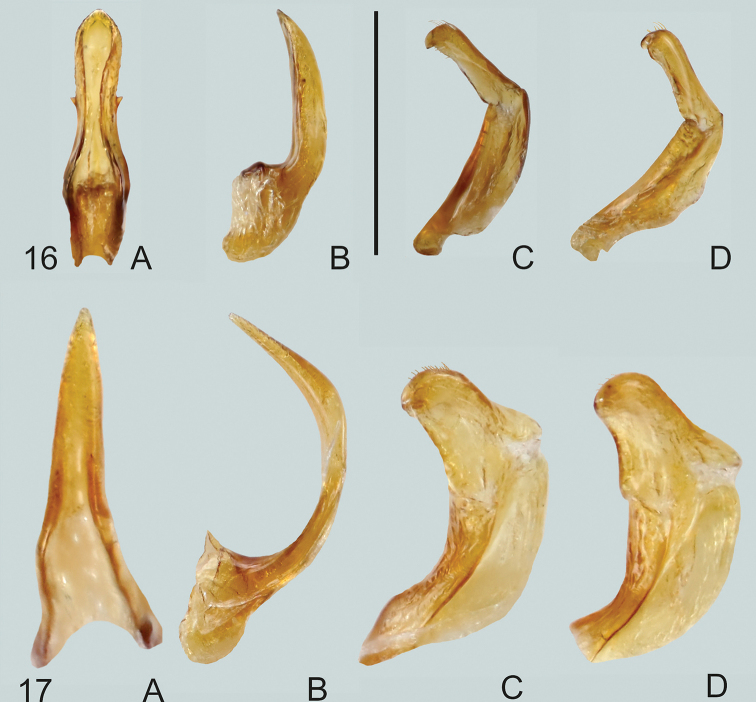
**16***Gibbidessus davidi* sp. nov. **17***G. drikdrikensis* sp. nov. **A** median lobe in ventral view **B** median lobe in lateral view, left side **C** left paramere in lateral view, **D** right paramere in lateral view. Scale bar: 0.2 mm.

***Ventral side*:** Ferruginous. Prothorax and apex of abdomen paler than other parts. Metacoxae and metaventrite covered with numerous larger punctures, surface shiny, without microreticulation. Abdominal ventrites with finer punctures, shiny, microreticulation absent. Metacoxal lines almost straight, anteriorly slightly divergent. Epipleuron ferruginous, coarsely punctate, shiny, lacking microsculpture. Legs ferruginous with meta-/mesotibia and meta-/mesotarsi somewhat darkened.

**Figures 18, 19.**
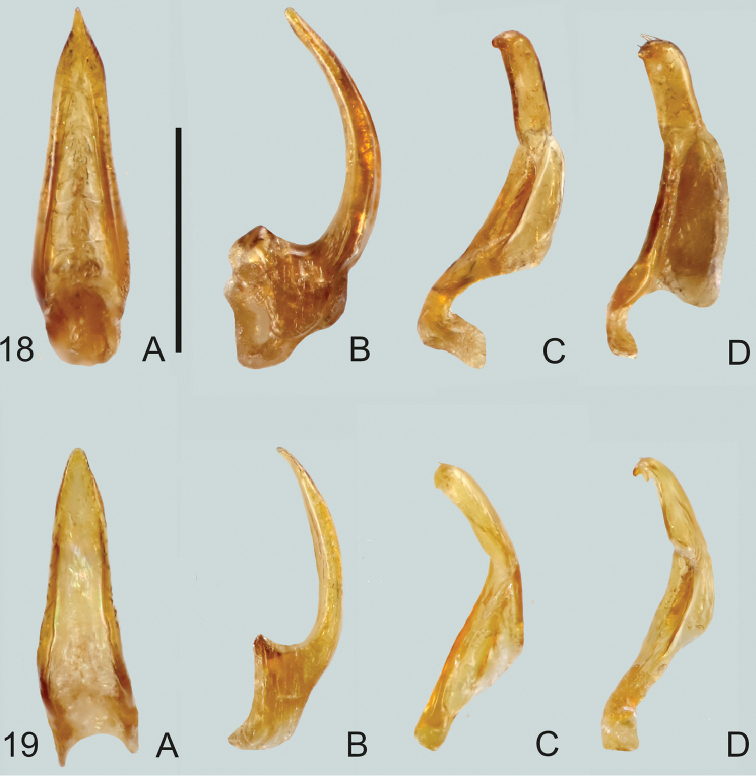
**18***Gibbidessus kangarooensis* sp. nov. **19***G. pederzanii* sp. nov. **A** median lobe in ventral view **B** median lobe in lateral view, left side **C** left paramere in lateral view **D** right paramere in lateral view. Scale bar: 0.2 mm.

**Figures 20, 21.**
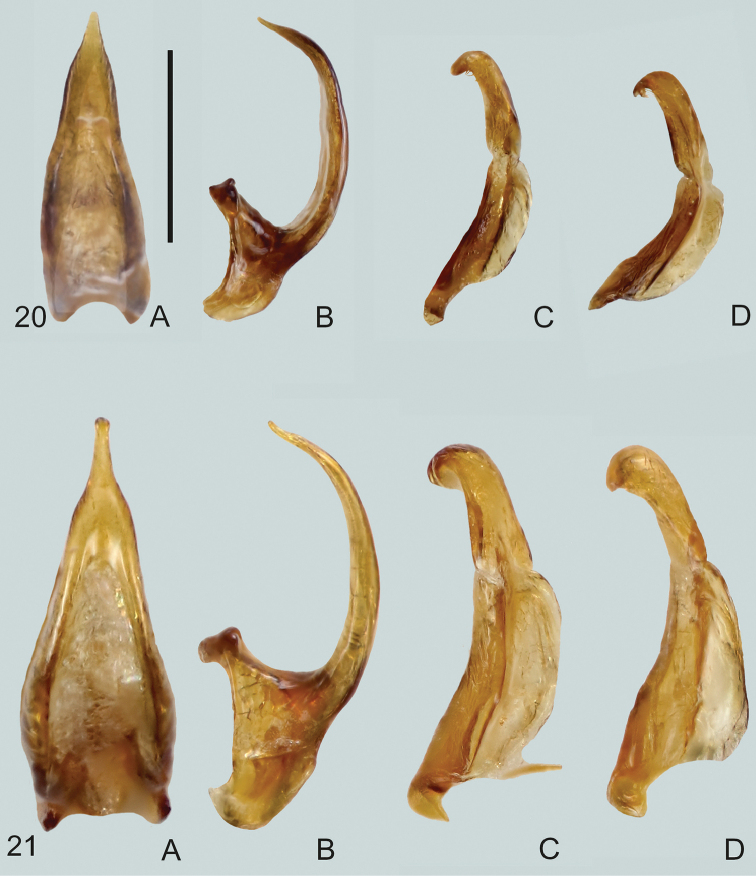
**20***Gibbidessus pictipes***21***G. rottnestensis* sp. nov. **A** median lobe in ventral view **B** median lobe in lateral view, left side **C** left paramere in lateral view, **D** right paramere in lateral view. Scale bar: 0.2 mm.

***Male*.** Dorsal surface with coarse punctures but otherwise with shiny surface (Fig. 8). Median lobe of aedeagus as in Fig. 20A, B. Shape of median lobe, bent evenly and fairly uniform in lateral view, in ventral view tapering and pointed at apex. Parameres bi-segmented, elongated, and with setae inside apical hook (Fig. 20C, D).

***Female*.** Dorsal surface almost mat, with coarse punctures and dense microreticulation (Fig. 9).

##### Affinities.

This species is similar to *G. kangarooensis* sp. nov. and the larger *G. rottnestensis* sp. nov. (TL = 1.5–1.7 mm) but readily separated by the different colour pattern. Furthermore, all three species can be separated by the form of their median lobes and parameres (Figs 18, 20, 21).

##### Distribution.

South-western Australia. The most common and widespread species in south-western Australia and a more inland species. South of a line from 230 km north of Perth (Leeman) to Albany (Fig. 24).

##### Habitat.

Permanent and seasonal, very shallow, sun exposed or half-shaded sedge swamps, lakeshores, larger ponds and flooded meadows on sandy bottom, with a thin layer of peat or rotten debris of sedges (Figs 30, 31). A winter and early spring breeder. Most specimens were collected in September and October, with the next generation in December and January. Apart from *G. pictipes*, the water beetle coenosis at Nalyerin Lake (Fig. 31) included the following species: Dytiscidae: *Limbodessus inornatus*, *Antiporus hollingsworthi*, *Sternopriscus minimus*, *Exocelina ater*; Hydrophilidae: *Enochrus eyrensis*, *Limnoxenus zealandicus*, *Paracymus pygmaeus* (see Hendrich 2001b). At Manypeaks (Fig. 30) *G. pictipes* was collected with the Dytiscidae: *Limbodessus inornatus*, *Sternopriscus browni* Sharp, 1882, *S. multimaculatus* (Clark, 1862), *S. storeyi* Hendrich & Watts, 2004, *S. wattsi* Hendrich & Watts, 2004, *Necterosoma darwinii* (Babington, 1841), *Rhantus suturalis* and *Lancetes lanceolatus* (Clark, 1863).

#### Gibbidessus rottnestensis sp. nov.

Taxon classificationAnimaliaColeopteraDytiscidae

F1C213AF-FE24-5ABE-AEA3-A8A92B7C2758

http://zoobank.org/C19B9505-4465-4C4B-BFD4-1BE35B7E645E

Figs 10 , 11 , 13 , 21 , 25 , 28 , 29

Uvarus pictipes (Lea, 1899): Watts (1978: 33, partim).

##### Type locality.

Australia, south-western Australia, Rottnest Island [32°0'22S, 115°30'26E].

##### Type material.

***Holotype*:** Male, “W AUS, ca. 25 km N Augusta on Rd. 250, shallow pool, 4.11.2013, leg. Wewalka (A4)” “Holotype *Gibbidessus rottnestensis* Hendrich, Watts & Balke des. 2020” [red printed label] (WAM). ***Paratypes* (27 exs.):** 4 specimens with same data as holotype (CGW, ZSM); 3 males, 1 female “Rottnest Is. [32°0'22S, 115°30'26E] Oct´ 31 W.A.”, “Australia, Harvard Exp., Darlington”, “Museum of Comparative Zoology”, “ANIC Database No. 25013255” (ANIC); 3 specimens with same data, and “SAMA Database No 25-00/596” (SAMA). 2 exs., “Australia, WA, Perth, Success, Beeliar RP, shallow peaty puddle 32°8'4S, 115°50'22E 21.-31.10.2015 L. Hendrich (WA 1/15)”, “M. Balke 7248”, “M. Balke 7247” [green, printed label] (ZSM); 1 ex., “Australia, WA, Albany Hwy, Muir Lakes Nature Reserve, SW part of Byenup Lagoon, 4. & 5.1.2000, 34°29'S, 116°44'E [34°30'4S, 116°44'19E], Hendrich leg. (loc. WA 11/157)” (CLH); 3 exs., “Australia, WA, 1 km W Kodjinup NP 34°24.03S, 116°38.37E [34°24'1S, 116°38'22E] 4.X.2003, CHS, Watts leg.”, one specimen “M. Balke 3921” [green, printed label] (CLH, SAMA); 1 ex., “WA Kodjinup N.R. [34°23'10S, 116°39'30E] 21/9/00 C.H.S.Watts”, “SAMA Database No 25-00/594” (SAMA); 2 exs., “AUSTRALIA, WA, Midlands, 38 Km ESE Cervantes, Wongonderrah Road, Nambung River Crossing, 9.9.2002, 30°33'21S, 115°21'27E, Hendrich leg. /Loc. 28b/192b” (CLH); 1 ex., “SW Australia/ N Bunbury, Yalgorup N.P. östl. Preston Beach [32°52'35S, 115°40'6E], 0m, 24.11.1996, Hendrich leg./Lok. 30” (CLH); 1 ex., “Australia, WA, RVDLE03 Riverdale Wetland [32°59'22S, 115°47'7E], 23/09/2008, South West Catchment Council Mon.” (DPAW); 1 ex., “Australia, WA, Pindicup Lake [34°24'35S, 116°43'20E], MUB030, 29.09.2014, Muir-Byenup Survey, M. Pennifold leg.” (DPAW); 1 ex., “Australia, WA, Pindicup Lake [34°24'35S, 116°43'20E], MUB030, 25.09.2014, Muir-Byenup Survey, M. Pennifold leg.” (DPAW); 2 ex., “Australia, WA, wetland north of Mialla Lagoon [33°10'04S, 115°44'01E], MIAL01, 8/10/2007, South-west Catchment Council Wetland Monitoring” (DPAW). All paratypes are provided with red printed paratype labels.

##### Note.

Watts (1978: 33) reports *Uvarus pictipes* from Rottnest Island (housed in Museum of Comparative Zoology and SAMA); these specimens belong to *Gibbidessus rottnestensis* sp. nov.

##### Diagnosis.

Larger species which externally is characterised by a more elongate body, shiny non-microreticulate dorsal surface, testaceous markings on elytra, and with distinct habitus disruption between pronotum and elytron. Dorsoventrally slightly flattened. Without cervical line but rather a few punctures instead (Fig. 10).

##### Measurements.

Holotype: TL = 1.85 mm, TL-H = 1.65 mm, MW = 0.95 mm. Paratypes: TL = 1.7–1.9 mm, TL-H = 1.5–1.7 mm, MW = 0.85–1.0 mm.

***Head*:** Black to ferruginous, without cervical line but rather a few punctures instead (punctures not obvious in females) (Fig. 13B). Evenly and coarsely punctate, shiny but with weak microreticulation. Punctures weak anteriorly and stronger posteriorly between eyes. Antennae relatively short, stout. Antennomeres 1-3 ferruginous, 4-11 darkened anteriorly.

***Pronotum*:** Ferruginous, anterior and posterior margins darker. Broadest at middle. Punctation very strong, almost evenly distributed, shiny and microsculpture absent. Sides of pronotum broadly margined and almost evenly rounded. Angle between pronotum and elytra well pronounced, basal pronotal plicae present. Striae moderately defined, almost 1/2 length of pronotum, strongly incurved.

***Elytra*:** Dark brown to black, with distinct basal and subapical testaceous markings (Fig. 10). Coarsely and densely punctate, shiny, microsculpture absent. Striae weakly impressed, slightly incurved and of same length as basal pronotal striae.

***Ventral side*:** Ferruginous. Prothorax and apex of abdomen paler than other parts. Metacoxae and metaventrite covered with numerous larger punctures, surface shiny, without microreticulation. Abdominal ventrites with finer punctures, shiny, microreticulation absent. Metacoxal lines almost straight, anteriorly slightly divergent. Epipleuron ferruginous, coarsely punctate, shiny, lacking microsculpture. Legs ferruginous with meta-/mesotibia and meta-/mesotarsae somewhat darkened.

***Male*.** Dorsal surface with coarse punctures but otherwise with shiny surface (Fig. 10). Median lobe of aedeagus as in Fig. 21A, B. Shape of median lobe, bent evenly and fairly uniform in lateral view, in ventral view strongly tapering and rounded at apex. Parameres bi-segmented, elongated, without setae inside apical hook (Fig. 21C, D).

***Female*.** Dorsal surface almost mat, with coarse punctures and dense microreticulation (Fig. 11).

##### Affinities.

This species is similar to the smaller *G. pictipes* (TL = 1.45–1.6 mm) but readily separated by the different colour pattern on elytra. Furthermore, both species can be separated by the form of their median lobes and parameres (Figs 20, 21).

##### Etymology.

The species is named after the type locality. The specific epithet is a substantive in the genitive case.

**Figures 22, 23.**
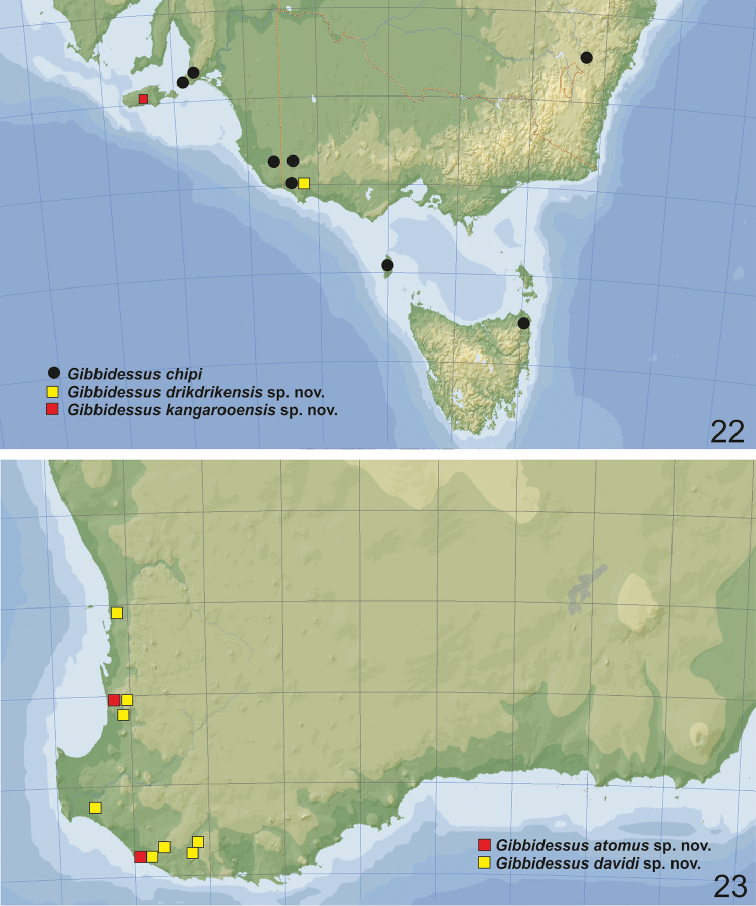
Distribution of **22***Gibbidessus chipi* (black dots), *G. drikdrikensis* sp. nov. (yellow square), *G. kangarooensis* sp. nov. (red square) **23***G. atomus* sp. nov. (red square), *G. davidi* sp. nov. (yellow square).

##### Distribution.

South-western Australia. Widespread but always rare and in low population densities. A more coastal species, from around 100 km north of Perth to south of Augusta and eastwards to the Muir Lakes (Fig. 25).

**Figures 24, 25.**
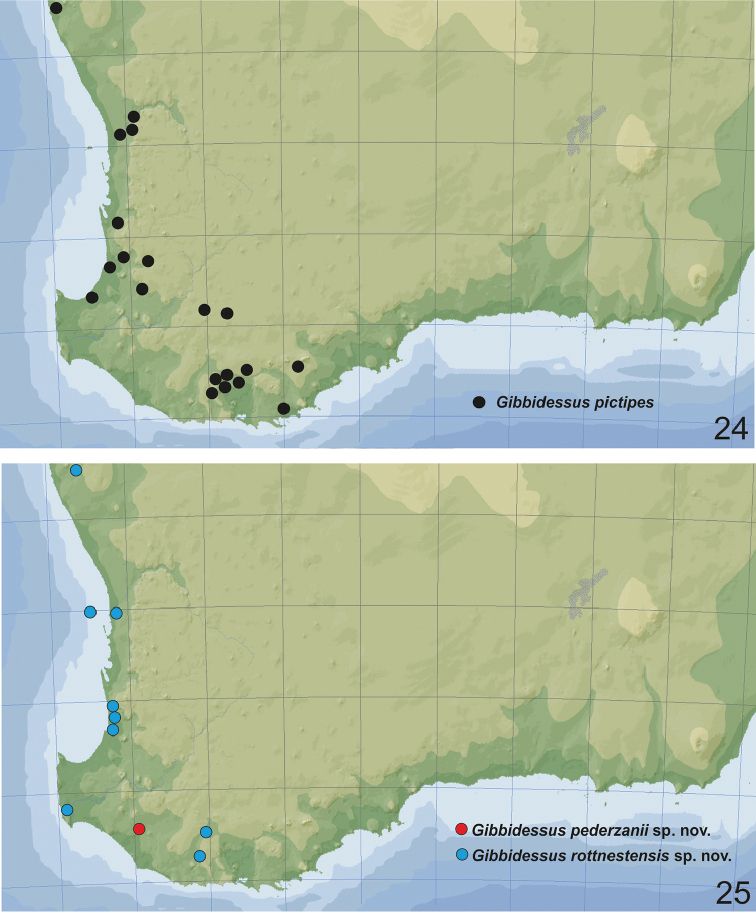
Distribution of **24***Gibbidessus pictipes* (black dots) **25***G. pederzanii* sp. nov. (red dot), *G. rottnestensis* sp. nov. (blue dots).

##### Habitat.

Seasonal, very shallow and exposed sedge swamps, pool and puddles on sandy bottom, with a thin layer of peat and rotten debris of sedges (Figs 28, 29). *Gibbidessus rottnestensis* sp. nov. tolerates slightly saline water as it was found at Preston Beach in a shallow lagoon near the coast. According to the data it is an early spring breeder. Most specimens were collected in September and October. In the Riverdale Wetland the species was syntopic with *Gibbidessus atomus* sp. nov. and *G. davidi* sp. nov. For the rich water beetle coenosis in Beeliar Regional Park near Perth see under *Gibbidessus davidi* sp. nov. In the seasonal swamps at Wongonderrah Road, near Nambung River Crossing, the species was collected with several hundred specimens of an undescribed *Exocelina* species and *Hyderodes crassus* Sharp, 1882; at Preston Beach north of Bunbury it was collected with *Hyphydrus elegans* (Montrouzier, 1860), *Necterosoma darwinii*, and *Platynectes aenescens* Sharp, 1882.

**Figure 26.**
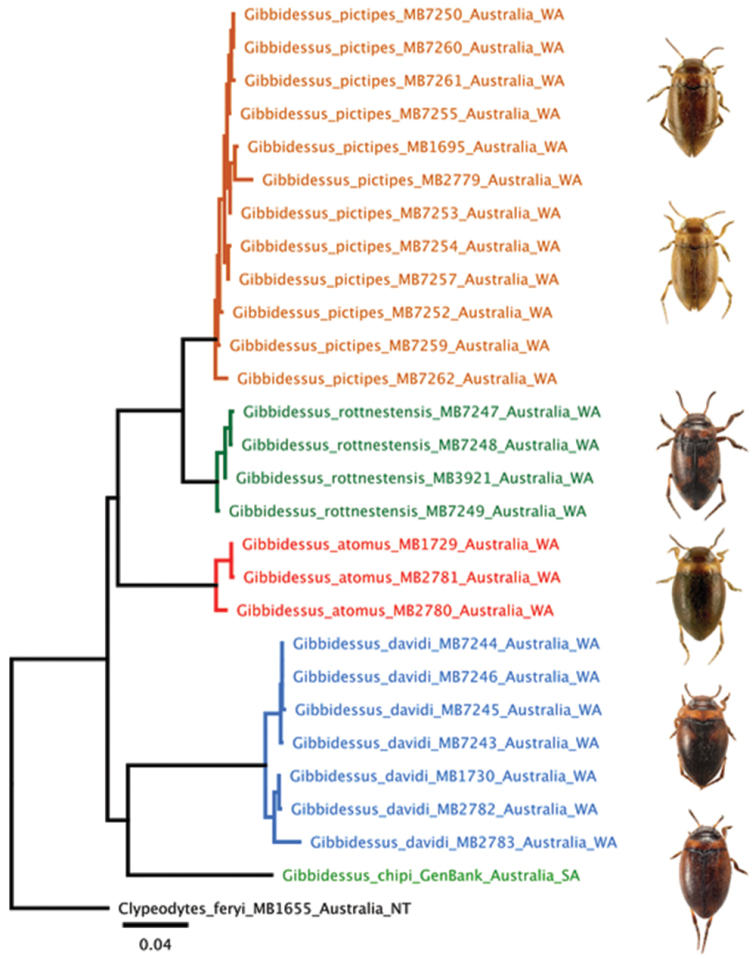
Maximum likelihood tree for Australian *Gibbidessus*. Neighbour joining tree (p-distances) calculated with Geneious (11.0.4.) software.

**Figure 27.**
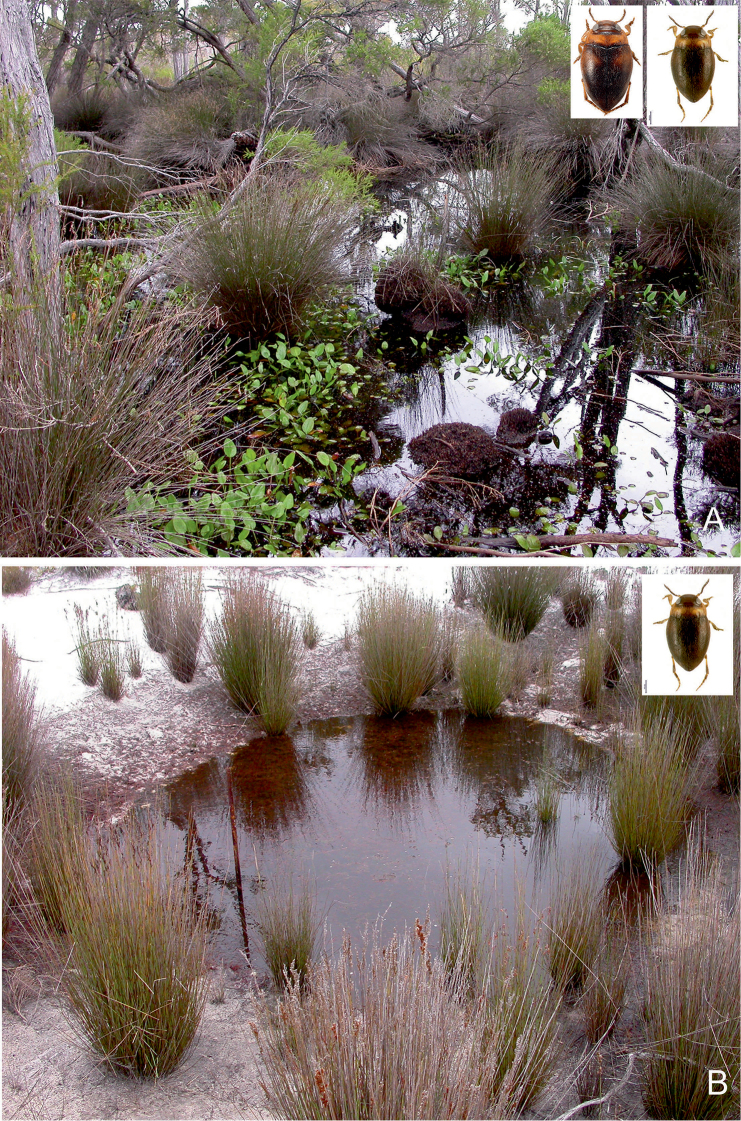
Habitat of *Gibbidessus atomus* sp. nov. and *Gibbidessus davidi* sp. nov. **A** Seasonally flooded *Melaleuca* sedge swamp **B** small and shallow heathland pool along Windy Harbour Road, south of Northcliffe, south-western Australia.

**Figure 28.**
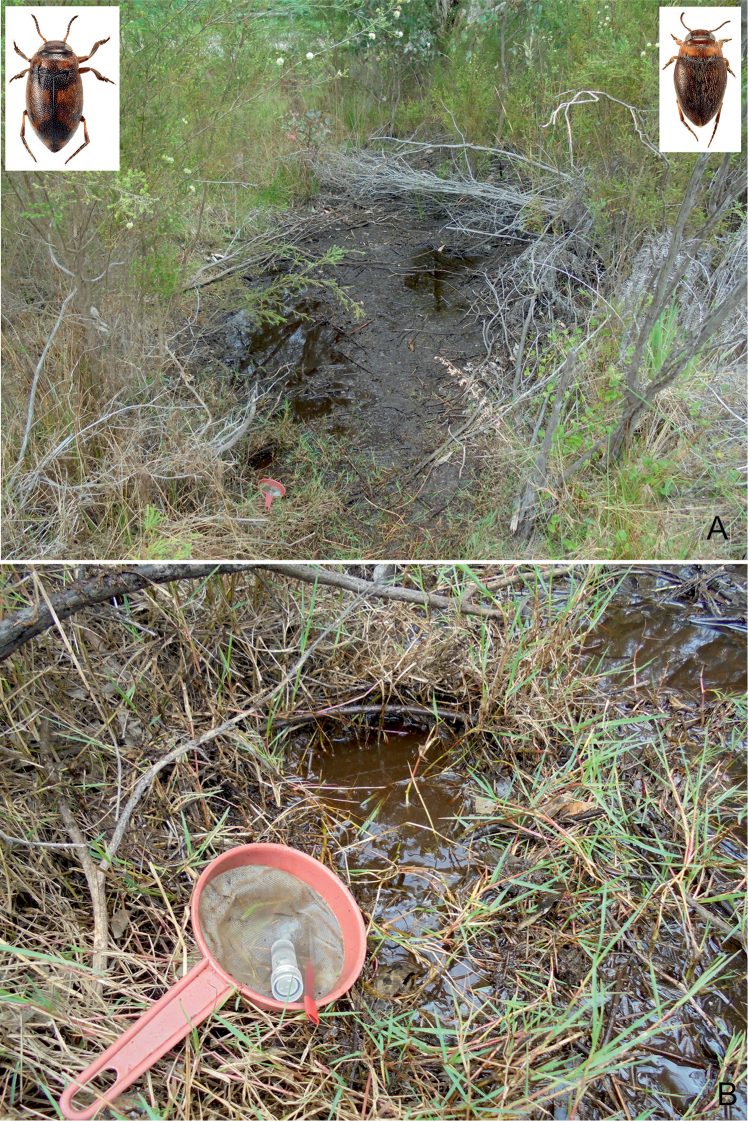
Habitat of *Gibbidessus davidi* sp. nov. and *Gibbidessus rottnestensis* sp. nov. **A** Shallow peaty pool with mats of floating grasses along Beeliar Swamps in Perth **B** same spot and habitat details with collecting methods.

**Figure 29.**
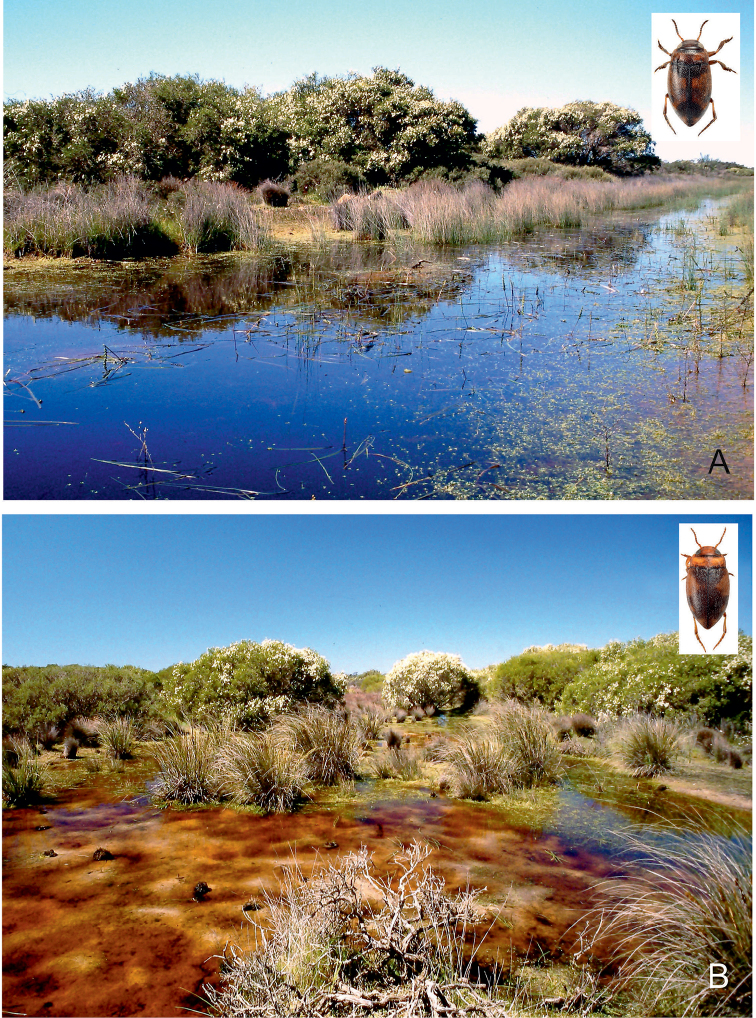
Habitat of *Gibbidessus rottnestensis* sp. nov. **A** and **B** seasonally flooded wetlands at Wongonderrah Road, Nambung River Crossing, 38 Km ESE Cervantes.

**Figure 30.**
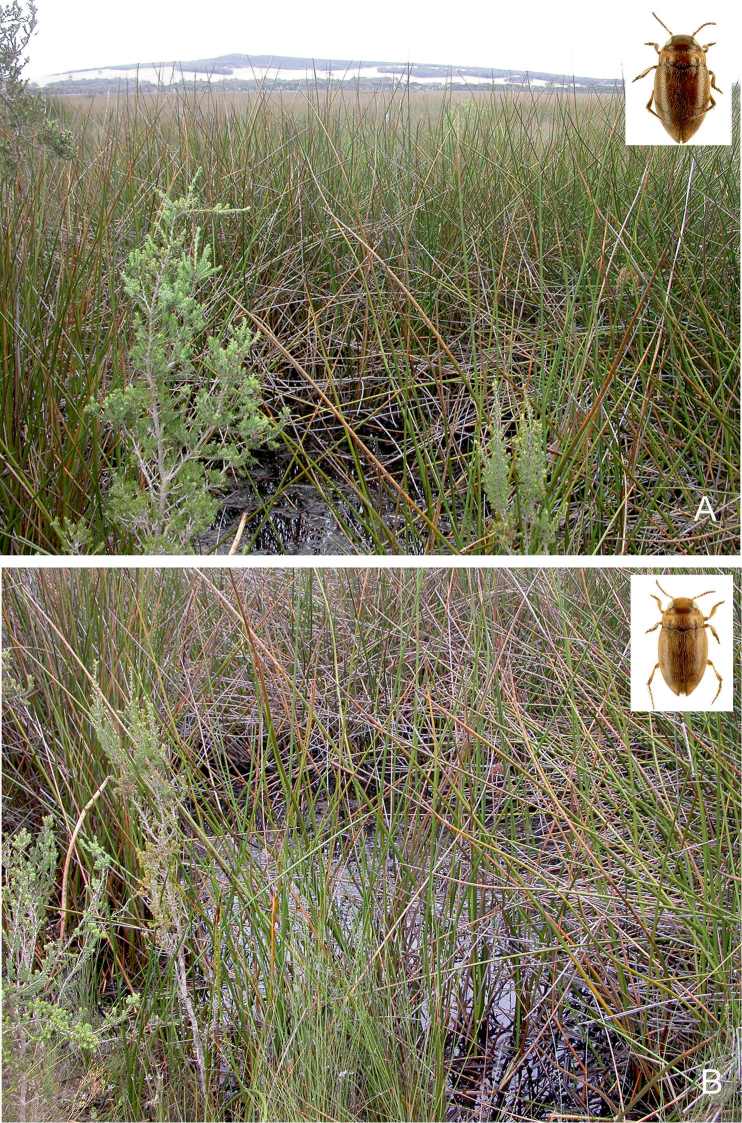
Habitat of *Gibbidessus pictipes*. **A, B** Seasonally flooded and exposed wetland with *Baumea* and sedges 3 km ENE Manypeaks, Lake Pleasant Nature Reserve.

**Figure 31.**
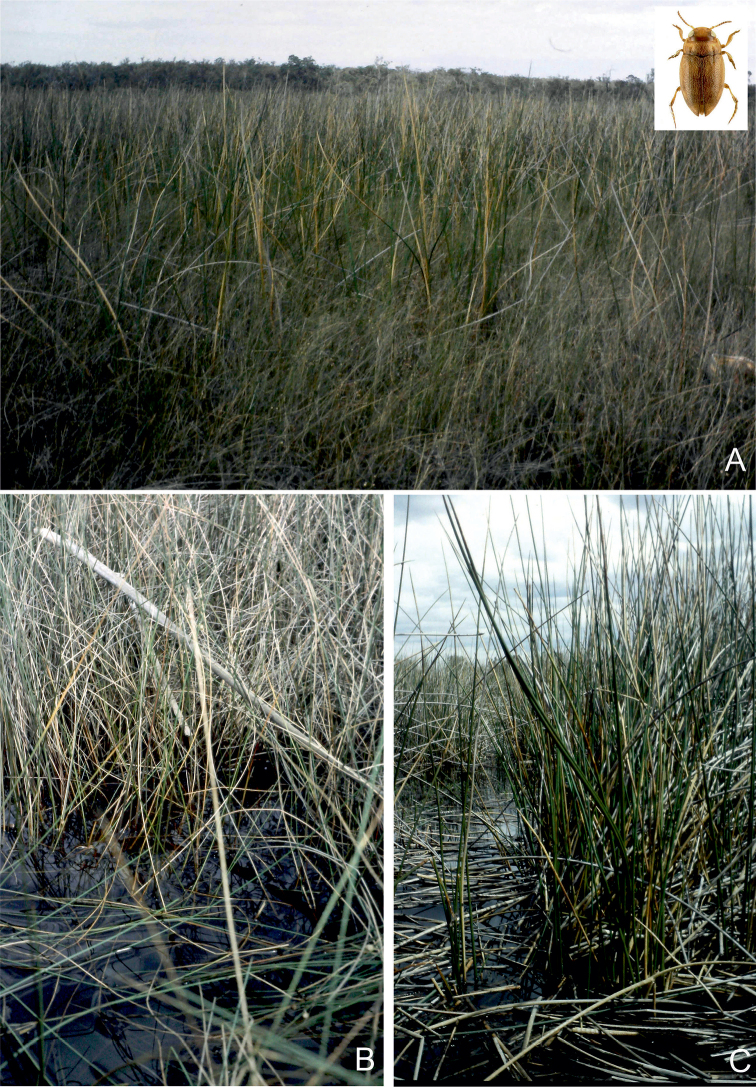
Habitat of *Gibbidessus pictipes*. **A, B** Seasonally flooded sedge swamp around lake Nalyerin **C** deeper and more permanent part of the lake, with stands of *Baumea*.

### Key to *Gibbidessus* Watts, 1978

**Table:** 

1	Head with cervical line (Fig. 12A). Body roundish and without habitus disruption between pronotum and elytron. Dorsoventrally rather domed	**2**
–	Head without cervical line (Figs 12B, 13). Body roundish or elongate, with or without habitus disruption between pronotum and elytron. Dorsoventrally domed or flattened	**5**
2	Species distributed in south-eastern Australia	**3**
–	Species distributed in south-western Australia	**4**
3	Smaller, TL = 1.5–1.55 mm. Median lobe and paramere as in Fig. 15. South Australia, Victoria, New South Wales, Tasmania	*** chipi ***
–	Larger, TL = 1.6–1.7 mm. Median lobe and parameres as in Fig. 17. Victoria	***drikdrikensis* sp. nov.**
4	Smaller, TL = 1.15–1.3 mm. Median lobe and paramere as in Fig. 12. From Perth area south to Northcliffe	***atomus* sp. nov.**
–	Larger, TL = 1.35–1.5 mm. Median lobe and parameres as in Fig. 16. From Perth area south to Northcliffe	***davidi* sp. nov.**
5	Species distributed in south-eastern Australia. Body elongate, with pronounced habitus disruption between pronotum and elytron. Dorsoventrally rather flattened, TL = 1.55 mm. Median lobe and parameres as in Fig. 18. Kangaroo Island, South Australia	***kangarooensis* sp. nov.**
–	Species distributed in south-western Australia	**6**
6	Body elongate, with pronounced habitus disruption between pronotum and elytron.	**7**
–	Body roundish and without habitus disruption between pronotum and elytron. Dorsoventrally rather domed, with more widely separated punctation on elytra. TL = 1.5–1.6 mm. Median lobe and parameres as in Fig. 19. Nannup and Pemberton area	***pederzanii* sp. nov.**
7	Elytron dark brown without distinct testaceous basal marking (Figs 8, 9). Smaller, TL = 1.45–1.6 mm. Median lobe and parameres as in Fig. 20. Occurs more inland, south of a line from Perth to Albany	***pictipes* comb. nov.**
–	Elytron with a broad testaceous basal marking (Figs 10, 11). Larger, TL = 1.7–1.9 mm. Median lobe and parameres as in Fig. 21. A more coastal species, from around 100 km north of Perth to south of Augusta and eastward to the Muir Lakes	.***rottnestensis* sp. nov.**

## Discussion

South-western Australia has long been recognised as a hotspot of aquatic macroinvertebrate and microfaunal diversity (Horwitz 1997; Segers and Shiel 2003). Four of the six described species of *Gibbidessus* are elements of this endemic freshwater fauna, and constitute a significant qualitative contribution to the biodiversity of the region. Three species are distributed in the southeast, including one endemic species from Kangaroo Island. Most probably more intensive studies will reveal further species occurring along the lowland coastal areas of south-western and south-eastern Australia.

Seven species of the genus are strictly lentic, appearing to be restricted to shallow and temporary pools, puddles, flooded meadows and seasonal sedge swamps in peatland areas or to very shallow waters at the edges of peaty lakes. One species, *G. pederzanii* sp. nov. was collected only at the edge of a shallow and slow-flowing forest creek. Occasionally, single specimens of *G. pictipes* and *G. chipi* have been found in slowly flowing or intermittent creeks. All species can be found in spring and early summer, and the majority of specimens have been collected between September and October. In the southern and more humid parts of south-western Australia, specimens of the new generation can be collected from November until January. Within any of their habitats, up to three species of the genus can be found (e.g., Riverdale Wetland); aggregations of several hundred specimens of at least one species are possible (e.g., Beeliar Regional Park in Perth and Lake Nalyerin). According to our experience, the occurrence of any *Gibbidessus* species indicates a high conservation value of the sampled water body or wetland.

The larvae of all species are still undescribed. The adults of all species seem to be capable of flight, but no specimens of any species have been obtained by operating light traps.

## Supplementary Material

XML Treatment for Gibbidessus

XML Treatment for Gibbidessus atomus

XML Treatment for Gibbidessus chipi

XML Treatment for Gibbidessus davidi

XML Treatment for Gibbidessus drikdrikensis

XML Treatment for Gibbidessus kangarooensis

XML Treatment for Gibbidessus pederzanii

XML Treatment for Gibbidessus pictipes

XML Treatment for Gibbidessus rottnestensis

## References

[B1] Balke M Ribera I (2004) Jumping across Wallace´s line: *Allodessus* and *Limbodessus* revisited (Coleoptera: Dytiscidae, Bidessini) based on molecular-phylogenetic and morphological data. Australian Journal of Entomology 43: 114–128. 10.1111/j.1440-6055.2004.00415.x

[B2] Balke M Warikar E Toussaint EFA Hendrich L (2013) *Papuadessus baueri* sp. nov. from Biak Island, Papua (Coleoptera: Dytiscidae: Hydroporinae). Spixiana 36(2): 283–288.

[B3] Balke M Ruthensteiner B Warikar E Neven K Hendrich L (2015) Two new species of *Limbodessus* diving beetles from New Guinea – short verbal descriptions flanked by online content (digital photography, μCT scans, drawings and DNA sequence data). Biodiversity Data Journal 3: e7096. 10.3897/BDJ.3.e7096 PMC4700388 26752969

[B4] Biström O (1988) Generic review of the Bidessini (Coleoptera, Dytiscidae). Acta Zoologica Fennica 184: 1–41.

[B5] Davies PE Brown K Walker R Cook L (2003) The aquatic fauna of King Island´s streams and wetlands. In: Donaghey R (Ed.) The Fauna of King Island. A Guide to Identification and Conservation Management. Natural Heritage Trust, Australia, 152 pp.

[B6] Hendrich L (2001a) A new species of *Antiporus* Sharp, 1882 from peatland swamps of south-western Australia (Coleoptera: Dytiscidae). Linzer biologische Beiträge 33(1): 299–308.

[B7] Hendrich L (2001b) A new species of *Hygrobia* Latreille, from peatlands of south-western Australia (Coleoptera: Hygrobiidae). Koleopterologische Rundschau 71: 17–25.

[B8] Hendrich L Balke M (2009) *Kakadudessus tomweiri*, a new genus and species of diving beetle from tropical northern Australia, based on molecular phylogenetic and morphological data (Coleoptera, Dytiscidae, Bidessini). Zootaxa 2134: 49–59. 10.11646/zootaxa.2134.1.4

[B9] Hendrich L Wang LJ (2006) Taxonomic revision of Australian *Clypeodytes* (Coleoptera: Dytiscidae, Bidessini). Entomological Problems 37(2): 1–11.

[B10] Hendrich L Pons J Ribera I Balke M (2010) Mitochondrial Cox1 Sequence Data Reliably Uncover Patterns of Insect Diversity but Suffer from High Lineage-Idiosyncratic Error Rates. PloS ONE 5(12): e14448. 10.1371/journal.pone.0014448 PMC3010977 21203427

[B11] Hendrich L Lemann C Weir TA (2019) 11. Dytiscidae LEACH, 1815. In: Slipinski A Lawrence J (Eds) Australian Beetles, Volume 2 – Archostemata, Myxophaga, Adephaga, Polyphaga (part).CSIRO Publishing, 34–60.

[B12] Horwitz P (1997) Comparative endemism and richness of the aquatic invertebrate fauna in peatlands and shrublands of far south-western Australia. Memoirs of the Museum of Victoria 56(2): 313–321. 10.24199/j.mmv.1997.56.19

[B13] Lawrence JF Weir TA Pyke JE (1987) Haliplidae, Hygrobiidae, Noteridae, Dytiscidae and Gyrinidae. In: Walton DW (Ed.) Zoological Catalogue of Australia. 4. Coleoptera: Archostemata, Myxophaga and Adephaga edited by the Bureau of Flora and Fauna. Canberra: Australian Government Publishing Service, 444 pp.

[B14] Lea AM (1899) Descriptions of new species of Australian Coleoptera. Part V. Proceedings of the Linnean Society of New South Wales 23: 512–645.

[B15] Miller KB Nilsson AN (2003) Homology and terminology: Communicating information about rotated structures in water beetles. Latissimus 17: 1–4.

[B16] Miller KB Short AEZ (2015) *Belladessus* Miller & Short (Coleoptera: Dytiscidae: Hydroporinae: Bidessini), new genus for two new species from northern South America: Parthenogenetic diving beetles? The Coleopterists Bulletin 69: 498–503. 10.1649/0010-065X-69.3.498

[B17] Nilsson AN Hájek J (2020) A World Catalogue of the Family Dytiscidae, or the Diving Beetles (Coleoptera, Adephaga). Version 1.I.2020. Distributed as a PDF file via Internet. http://www.waterbeetles.eu [accessed 08 February 2020]

[B18] Segers H Shiel RJ (2003) Microfaunal Diversity in a Biodiversity Hotspot: New Rotifers from Southwestern Australia. Zoological Studies 42(4): 516–521.

[B19] Watts CHS (1978) A revision of the Australian Dytiscidae (Coleoptera). Australian Journal of Zoology Supplement Series 57: 1–166. 10.1071/AJZS057

[B20] Watts CHS (1985) A faunal assessment of Australian Hydradephaga. Proceedings of the Academy of Natural Sciences of Philadelphia 137(1): 22–28.

[B21] Watts CHS (2002) Checklist and guides to the identification, to genus, of adults and larval Australian water beetles of the families Dytiscidae, Noteridae, Hygrobiidae, Haliplidae, Gyrinidae, Hydraenidae and the superfamily Hydrophiloidea (Insecta – Coleoptera). Cooperative Research Centre for Freshwater Ecology (Australia). Identification and Ecology Guide 43: 1–110.

[B22] Watts CHS Humphreys WF (2001) A new genus and six new species of Dytiscidae (Coleoptera) from underground waters in the Yilgarn palaeodrainage system of Western Australia. Records of the South Australian Museum 34(2): 99–114.

[B23] Watts CHS Humphreys WF (2003) Twenty-five new Dytiscidae (Coleoptera) of the genera *Tjirtudessus* Watts & Humphreys, *Nirripirti* Watts & Humphreys and *Bidessodes* Régimbart from underground waters in Australia. Records of the South Australian Museum 36(2): 135–187.

[B24] Watts CHS Humphreys WF (2004) Thirteen new Dytiscidae (Coleoptera) of the genera *Boongurrus* Larson, *Tjirtudessus* Watts & Humphreys and *Nirripirti* Watts & Humphreys, from underground waters in Australia. Transactions of the Royal Society of South Australia 128(2): 99–129.

[B25] Watts CHS Humphreys WF (2006) Twenty-six new Dytiscidae (Coleoptera) of the genera *Limbodessus* Guignot and *Nirripirti* Watts & Humphreys, from underground waters in Australia. Transactions of the Royal Society of South Australia 130(1): 123–185. 10.1080/3721426.2006.10887055

[B26] Watts CHS Humphreys WF (2009) Fourteen new Dytiscidae (Coleoptera) of the genera *Limbodessus* Guignot, *Paroster* Sharp, and *Exocelina* Broun from underground waters in Australia. Transactions of the Royal Society of South Australia 133(1): 62–107. 10.1080/03721426.2009.10887112

[B27] Watts CHS Leys R (2005) Review of the epigean species of Australian *Limbodessus* Guignot (Insecta: Coleoptera: Dytiscidae). Transactions of the Royal Society of South Australia 129: 1–13.

